# Multiscale topology characterizes dynamic tumor vascular networks

**DOI:** 10.1126/sciadv.abm2456

**Published:** 2022-06-10

**Authors:** Bernadette J. Stolz, Jakob Kaeppler, Bostjan Markelc, Franziska Braun, Florian Lipsmeier, Ruth J. Muschel, Helen M. Byrne, Heather A. Harrington

**Affiliations:** 1 Mathematical Institute, University of Oxford, Oxford, UK.; 2 Oxford Institute for Radiation Oncology, University of Oxford, Oxford, UK.; 3 Department of Experimental Oncology, Institute of Oncology Ljubljana, Ljubljana, Slovenia.; 4 Data Science, pRED Informatics, Pharma Research & Early Development, Roche Innovation Center Munich, Munich, Germany.; 5 Digital Biomarkers, pRED Informatics, Pharma Research & Early Development, Roche Innovation Center Basel, Basel, Switzerland.; 6 Wellcome Centre for Human Genetics, University of Oxford, Oxford, UK.

## Abstract

Advances in imaging techniques enable high-resolution three-dimensional (3D) visualization of vascular networks over time and reveal abnormal structural features such as twists and loops, and their quantification is an active area of research. Here, we showcase how topological data analysis, the mathematical field that studies the “shape” of data, can characterize the geometric, spatial, and temporal organization of vascular networks. We propose two topological lenses to study vasculature, which capture inherent multiscale features and vessel connectivity, and surpass the single-scale analysis of existing methods. We analyze images collected using intravital and ultramicroscopy modalities and quantify spatiotemporal variation of twists, loops, and avascular regions (voids) in 3D vascular networks. This topological approach validates and quantifies known qualitative trends such as dynamic changes in tortuosity and loops in response to antibodies that modulate vessel sprouting; furthermore, it quantifies the effect of radiotherapy on vessel architecture.

## Teaser

Topological data analysis enables deep quantification of response to drug and radiation treatment in 3D tumor vasculature.

## INTRODUCTION

The advent of high-resolution imaging techniques has driven the development of reconstruction algorithms, which generate exquisitely detailed three-dimensional (3D) renderings of biological tissues, such as tumor vascular networks (*1*, *2*). Tumor vasculature is highly dysfunctional as vessels tend to be very leaky, the direction of blood flow can change over time, and the structure of the vessel network looks markedly different from that of normal vessels (*3*). Visualization of tumor vasculature in 3D and over time offers a detailed picture of abnormal structural changes such as twists and loops (*3*–*8*). The quantification of the 3D architecture is important because vessel structure affects vessel function (i.e., delivery of oxygen, nutrients, and therapies). Existing analyses have quantified structural features—including vessel density, number of vessels and branching points (*9*), fractal dimension (*10*), and lacunarity (*11*)—and highlighted their relevance for predicting disease progression (*12*, *13*) and response to treatment (*8*). Such spatially averaged summaries lose information from detailed 3D renderings and do not account for vessel connectivity or higher-order topological features such as loops and voids; the latter correspond to avascular tumor regions characterized by hypoxia and necrosis and associated with reduced patient survival and poor responses to therapy (*3*). Therefore, more detailed, automated, and reproducible methods for quantifying vessel networks are needed, which may provide future benefit to basic research, clinical assessments, treatment planning, and monitoring.

For large studies (*14*), machine learning algorithms are excellent at quantifying 3D microscopy features [e.g., images obtained from adipose tissue (*15*)]. Here, we use existing image processing methods, based on machine learning, to reconstruct 3D vascular networks from high-resolution spatial data. We then use these 3D segmented networks to quantify, compare, and interpret the spatial organization of tumor vasculature and responses to treatment. The novelty of our approach lies in the deep quantification of the vascular networks and not the collection and segmentation of the experimental data. In more detail, we present a topological framework that quantifies different notions of connectivity in reconstructed 3D vascular networks (e.g., quantifying loops and voids), complements, extends, and surpasses existing descriptors (see figs. S23 and S28) by providing a multiscale summary of these topological features.

Mathematically, one can describe tumor vasculature as a spatial network, i.e., nodes embedded in 3D space, connected by edges that represent blood vessel segments. An emerging mathematical field that uses topological and geometric approaches to quantify the “shape” of data is topological data analysis (TDA) (*16*, *17*). A central method in TDA is persistent homology (PH) (*16*–*20*). PH computes features called topological invariants of the data at different spatial scales; features that persist over a wide range of spatial scales are generally considered better to represent robust topological signals in the data. TDA has been successful in neuroscience, specifically analyzing functional brain network data [for a small selection of examples, see (*21*–*25*)]. Improved computations in PH (*20*) have increased the scope of its applications to include structural and spatial data, such as brain arteries (*26*), neurons (*27*), airways (*28*), stenosis (*29*), zebrafish patterns (*30*), contagion dynamics (*31*), and spatial networks (*24*, *32*–*34*). More recently, interest has emerged for applications of TDA to patient-specific data in oncology (*35*), and PH has been successfully applied to classify synthetic data from mathematical models of angiogenesis, the process in which tumor blood vessels form from existing ones (*36*). The characteristics of tumor vascular networks that we study here using PH features are tortuosity (*4*) (or “bendiness”), loops (*4*), and size of avascular regions (*3*).

Tortuosity has been quantified previously using standard measures in tumor vessels (*37*, *38*) and using TDA in aging vasculature (*26*). Here, we propose a normalized topological tortuosity descriptor. To our knowledge, this is the first time that loops and voids (which may correspond to avascular tumor regions) have been quantified in vasculature. The leap from a single 2D slice to 3D reconstruction presents opportunities for quantifying 3D connectivity that would otherwise be impossible. As we describe later, these topological approaches are inherently multiscale and quantify global connectivity of the data that surpasses standard descriptors. Such quantification could serve as a biomarker for characteristics of vascular networks and their response to vascular targeting treatments.

**Fig. 1.**
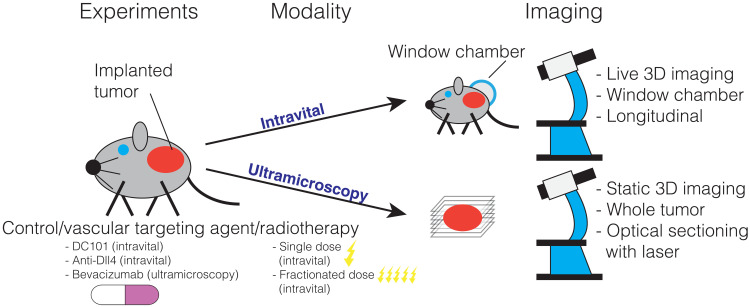
Description of datasets. We illustrate the treatments and imaging techniques used to generate the experimental data that we analyze. Both datasets consist of 3D stacks of tumor vasculature images from mice undergoing different treatments (vascular targeting agents and radiotherapy). Intravital data were collected from live animals observed over several days. Ultramicroscopy data (*8*) were obtained from multiple tumors excised at different times after treatment (one time point per tumor). These data are not directly comparable since they were generated from two different mouse models (see Table 1) using different experimental setups (see Materials and Methods).

**Fig. 2.**
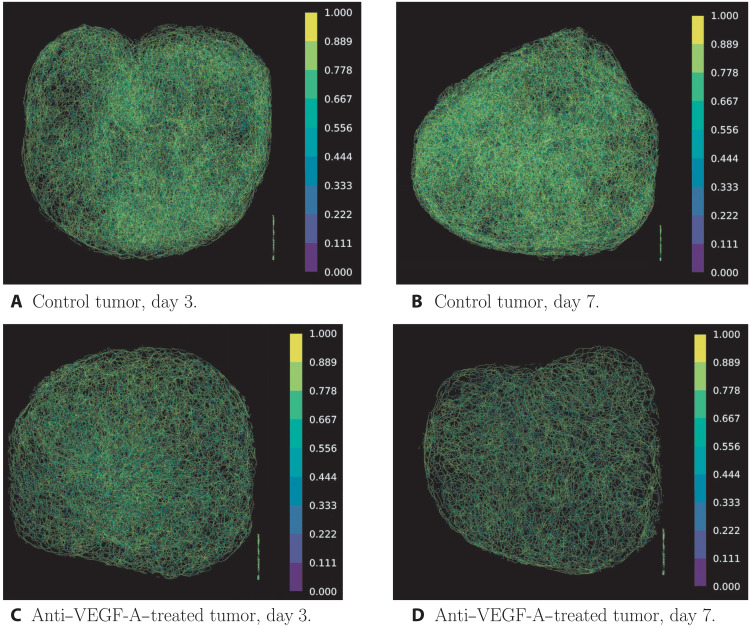
Example images of extracted vessel networks from multispectral fluorescence ultramicroscopy data colored according to tortuosity measured via clr values. We can see a clear difference between the vessel networks of the treated and the untreated tumor on both days 3 and 7 after treatment. Note that the collection of lines in the bottom right corner of the images corresponds to text that was present in the skeleton images in the dataset. We removed these artifacts from our extracted point clouds. (**A**) Control tumor, day 3. (**B**) Control tumor, day 7. (**C**) Anti–VEGF-A–treated tumor, day 3. (**D**) Anti–VEGF-A–treated tumor, day 7.

We showcase our topological approach by analyzing 3D vascular networks reconstructed from microscopy images from two different studies: intravital data and ultramicrospy data (*8*) (see Fig. 1 and corresponding table). In the intravital dataset, the same vascular networks are observed over time, providing a time course. The low penetration depth of intravital imaging means that only part of the vasculature can be imaged. The intravital dataset contains control (untreated) tumors and tumors subjected to either vascular targeting agents or radiation therapy. The agents consist of antibodies DC101 (*37*) and anti-Dll4 (*39*), which decrease and increase vessel sprouting, respectively (*9*, *12*, *40*, *41*). The irradiated tumors receive either a single dose [1 × 15 gray (Gy)] or fractionated doses (5 × 3 Gy) of radiation therapy. Although radiation therapy is commonly used to treat tumors, observations of structural changes in the vasculature have remained inconsistent (*42*). The second dataset, imaged using ultramicroscopy (*8*), gives 3D reconstructions of the entire tumor vasculature. The dataset includes multiple time points (snapshot data), where we obtain one time point per tumor. The data include control tumors and tumors treated with bevacizumab (*43*), a drug that inhibits angiogenesis and is thought to (transiently) normalize (*3*) tumor vasculature, i.e., reduce structural and functional abnormalities. See the “Data preprocessing” section in Materials and Methods for details on network binarization, skeletonization, pruning, and testing to reconstruct the networks that we analyze (*1*, *2*, *8*, *44*, *45*). We show example images of the vessel networks extracted from the ultramicroscopy dataset in Fig. 2.

**Table 1.** Summary of datasets. Summary of datasets analyzed in this study including the number of mice *n* (see also Fig. 1). For information on the size of the images and extracted networks, see table S1.

**Dataset**	**Type**	**Model**	**Experimental conditions**
Multiphoton intravital 3D microscopy	Dynamic (over multiple days)	Mouse colorectal cancer in mice	1. Control (*n* = 7)
			2. DC101 (decreases sprouting, *n* = 5)
			3. Anti-Dll4 (increases sprouting, *n* = 3)
			4. Irradiated (single-dose 15 Gy, *n* = 5)
			5. Irradiated (fractionated-dose 5 × 3 Gy, *n* = 4)
Multispectral fluorescence ultramicroscopy	Static	Human breast cancer in mice	1. Control (*n* = 18)
			2. Bevacizumab (*n* = 13)

### Standard measures and existing descriptors

To describe the architecture of abnormal tumor vasculature, several morphological characteristics have been used. The most common one, microvascular density (MVD), is often used to compare 2D tumor sections. A high MVD has been shown to independently predict death from several types of cancer (*46*). Other descriptors often used in the literature include vessel volume, number of branching points, vessel diameter, vessel length, and vessel tortuosity (*38*). However, none of these features can recapitulate the complexity of the entire vascular network. With the advent of personalized medicine, different imaging modalities such as magnetic resonance imaging (MRI), dynamic contrast–enhanced MRI, and computed tomography are often used to aid with patient diagnosis and treatment personalization. A common feature to these approaches is that the resulting images are 3D volumes (*37*). Hence, the morphological descriptors should capture the complexity of the 3D vascular network, not just the single-vessel-scale characteristics.

Existing analyses of blood vessel networks have quantified structural features and shape, including vessel density, number of vessels (i.e., number of edges), and branching points (i.e., number of nodes) (*9*). To highlight the additional insight generated by our TDA descriptors, we calculate existing descriptors (at each time point), specifically the number of branching points, mean vessel diameter, mean vessel length, and length-to-diameter ratio for both the intravital data and ultramicroscopy data. For the intravital data, we report two existing tortuosity measures (for details, see Materials and Methods). The first tortuosity descriptor is the chord-length ratio (clr) (*2*, *45*), which is the ratio of the distance between the branching or end points of the vessel and the path length of the vessel, where a value of one corresponds to a straight vessel and zero is tortuous. The second tortuosity descriptor is the sum-of-angles metric (SOAM) (*13*), which is computed by summing the angles of regularly sampled tangents along a blood vessel skeleton, where a value of zero corresponds to a straight vessel and tortuous vessels correspond to larger values. For both datasets, we calculate the number of vessel segments and number of branching points [both as computed in (*2*, *44*)]. For the ultramicroscopy data, we aditionally report the above descriptors as previously computed in (*8*) as well as necrotic tumor volume, tumor volume, and vital tumor volume all as computed in (*8*). All existing descriptors are normalized by day 0 of observation/treatment and computed from the freely available python code package unet-core (*44*). Note that to compare with existing descriptors, we compress our TDA descriptors and therefore lose information.

### Topological data analysis

Here, we present topological descriptors to quantify vascular network characteristics across different spatial scales and over time. We first explore appropriate multiscale lenses of the data, called filtrations, which feed into PH computations.

We propose two filtrations for tumor vascular networks: The radial filtration quantifies topological features with respect to distance from the tumor center; the α-complex (*47*) filtration (see the “Topological data analysis” section in Materials and Methods) quantifies avascular tumor regions that are devoid of blood vessels. Recall that the data are embedded in 3D space. The network nodes are branching points (i.e., points where vessels branch) and vessel nodes (i.e., other points sampled along vessels). In the radial filtration, we determine the center of mass of the 3D nodes and grow a sphere from the center outward in uniform steps. Inspired by a filtration to analyze neuronal tree morphologies radially from the root of a neuron (*27*), the radial filtration differs from the well-known Vietoris-Rips and Čech filtrations since we only consider one ball from the center of the tumor (rather than many balls, for example, grown from points sampled on the network). At each step, we determine the nodes located inside the growing sphere and connect two nodes when there is a vessel between them, resulting in a growing network, the radial filtration. We then compute the connected components and loops. As the data are 3D, loops that we find in this way are not artifacts of projections but genuine features of the vascular networks. The radial filtration depends on the choice of tumor center, whereas the α-complex filtration does not. Note that, in contrast to the aforementioned Vietoris-Rips and Čech filtrations as well as the α-complex, the radial filtration never fills in triangles and contains only nodes and edges inferred from the underlying biological network structure. For the α-complex filtration (*20*, *47*), we construct a sequence of nested simplicial complexes (i.e., collections of nodes, edges, triangles, and tetrahedra) on the 3D nodes of the vessel network. Each edge, triangle, or tetrahedron can be assigned a filtration value α^2^, which can be thought of as a proxy for volume. The filtration value is increased to obtain the filtration on the data until the Delaunay triangulation (*48*) of the 3D nodes of the vessel network is constructed. We then compute voids in this filtration.

**Fig. 3.**
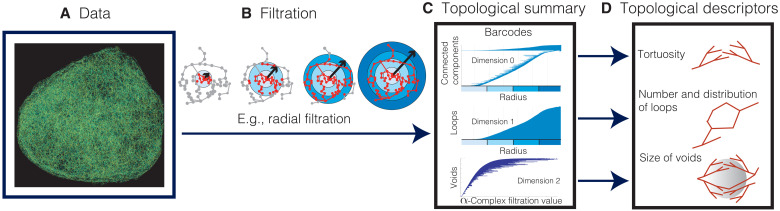
Schematic illustration of TDA for vascular network data. (**A**) We reconstruct the 3D vascular network from image stacks. (**B**) We apply the radial filtration and the α-complex filtration. (**C**) We compute the topological summary of the data, which consists of a collection of barcodes (*49*). The horizontal axis of a barcode represents a spatial parameter such as radial distance to the tumor center (radial filtration) or the scale at which we view the data (α-complex filtration). Every line in a barcode corresponds to a topological feature—i.e., a connected component, loop, or void—in the data. In the radial filtration, we analyze the network within the sphere (highlighted in red) and compute connected components and loops as the sphere grows from the tumor center outward. In the barcodes, the bars start at the radius (measured from the tumor center) where the corresponding connected component or loop first enters the sphere. For a connected component, its corresponding bar ends at the radius at which it merges with another component, i.e., it connects to another part of the vascular network within the growing sphere. A bar representing a loop finishes at the final radius of the filtration. For voids, we study the data at different scales using the α-complex filtration (see the “Topological data analysis” section in Materials and Methods), and the range of a bar represents the scale values where the void is detectable. Its length is a proxy for the volume of the void. (**D**) We extract interpretable topological descriptors of the data from barcodes.

PH computes topological features such as connected components (dimension 0), loops (dimension 1), and voids (dimension 2) and how they change across different scales. These multiscale and multidimensional topological features are summarized in a barcode (*49*) (see Fig. 3). From these barcodes, we compute interpretable topological descriptors in Results. These topological calculations extend the toolbox of existing descriptors by quantifying connectivity across spatial and temporal scales.

### Loss of information

Given the sheer size of these data (see the “Computational differences between datasets” section in the Supplementary Materials for the variation in size), they cannot be processed, analyzed, and summarized without some loss of information. At the processing stage, the reconstruction of 3D networks from 2D slices in the ultramicroscopy dataset (*8*) had original image stacks taken with a resolution of 5.1 μm, thus limiting the loss of information in the *z* direction while reconstructing the image stack. We used existing segmentation and skeletonization algorithms (*1*, *2*, *45*) and existing code that computes skeletonization and standard metrics (*2*, *44*) (see Materials and Methods for details on data, processing, and testing to minimize segmentation errors). Data analysis quantities depend on the segmentation and preprocessing of the data. For biological networks, noise contamination and its consequences on data analysis are an active area of research (*50*). A strength of TDA is that its output has been proven to be robust to small amounts of noise in data, which are given by stability theorems (*51*). However, the TDA output will change if the resulting network changes substantially, as will existing morphological descriptors. The TDA filtration step sizes are discrete and coarse to ensure that computations are feasible for this dataset (e.g., 500 filtration steps for the radial filtration); therefore, small features may be “stepped over,” and some fine information may be lost between filtration steps. As will be described later (see Results and the “Computational differences between datasets” section in the Supplementary Materials), the topological tortuosity measure depends on the short bars in the barcode. For the ultramicroscopy data, we may have required more filtration steps or finer data resolution in the *x*-*y* plane to compute topological tortuosity; however, we were limited by computational resource and processing of experimental data. Furthermore, because of the size of the reconstructed ultramicroscopy networks (e.g., ranging from 12,500 to 118,000 branching points; see the “Computational differences between datasets” section in the Supplementary Materials), we had to subsample points from the network (see the “Data preprocessing” section in Materials and Methods for details). All these factors may affect the computation of small connected components (see discussion of tortuosity descriptor in Results and the Supplementary Materials).

### Gain of information

The PH algorithm used for the TDA computations is underpinned by stability theorems (*51*), which ensure that the computed topological features are stable with respect to small perturbations to the data. Moreover, the algorithm will output the same topological barcode (summarizing the multiscale descriptors) even if it is rerun or computed multiple times on the same dataset; therefore, it is both accurate and reproducible. In contrast, manual counting is prone to human errors and is often limited to 2D slices, as done by Shayan *et al.* (*52*), limiting detection to features (e.g., vessel segments, branching points, and loops) in the plane. Rather than manual counting or standard descriptors, the mathematical framework that we use (theory and algorithms) enables quantification of loops and voids in 3D. Furthermore, this topological quantification is automated, systematic, and performed across spatial scales. To compare this multiscale TDA to standard descriptors (which are single scale) requires its compression; TDA gives additional information that surpasses manual counting and existing descriptors. Therefore, topological descriptors offer a substantial improvement on both manual counts and standard descriptors.

## RESULTS

### Topology gives descriptors of tortuosity, loops, and voids

**Fig. 4.**
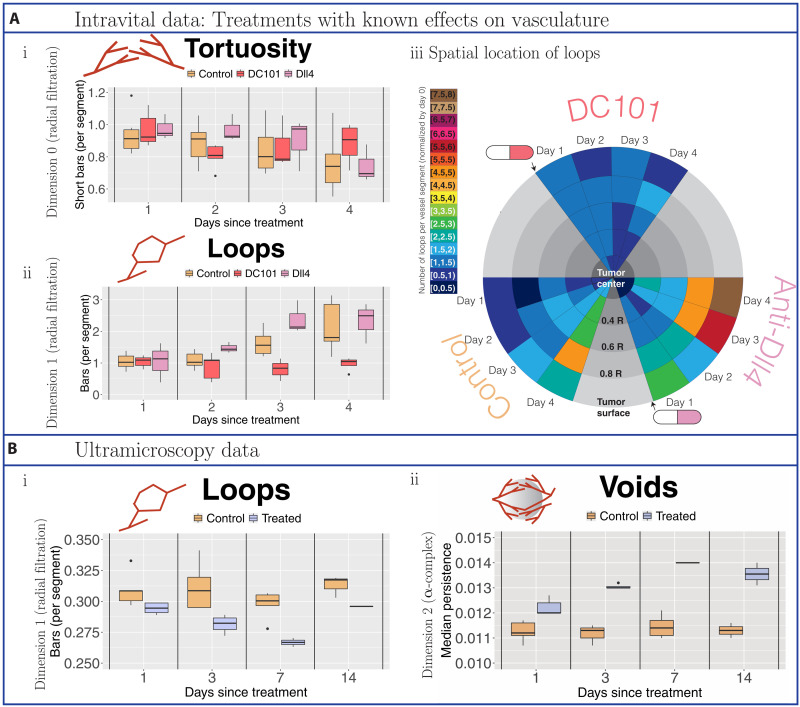
Topological descriptors extracted from tumor blood vessel networks treated with vascular targeting agents with known effects. (**A**) Intravital data results. We normalized all descriptors with respect to values on the day on which treatment is administered or, for controls, the day on which observations commence (day 0). Data were collected from controls (beige) and tumors treated with the vascular targeting agent DC101 (*37*) (dark pink) or the vascular targeting agent anti-Dll4 (*39*) (light pink). (i) Tortuosity was computed as the ratio of short bars in dimension 0 barcodes of the radial filtration (≤10% of maximal radius used) to the number of vessel segments. (ii) Loops are the number of bars in dimension 1 barcodes of the radial filtration per vessel segment. (iii) Spatiotemporal resolution of the number of loops per vessel segment. We illustrate the changes in the median number of loops (normalized by day 0) in radial intervals around the tumor centers over the days of observation. We point to the day following treatment with vascular targeting agents with a cartoon drug. (**B**) Ultramicroscopy data results. Because of the snapshot nature of the data (one time point per tumor), all reported topological descriptors are raw values. Data were collected from controls (beige) and tumors treated with bevacizumab (purple). (i) We computed the number of vessel loops per vessel segment. (ii) We determined the size of voids (avascular regions) by computing the median length of bars in the dimension 2 barcodes of the α-complex filtration.

We developed interpretable, quantitative descriptors of tortuosity (*26*) (bendiness), loops, and voids (see Figs. 3D and 4) based on the calculated topological summaries of 3D tumor vasculature. The connected components (dimension 0) of the radial filtration characterize the tortuosity: A vessel with high tortuosity will intersect the growing sphere multiple times and generate many small components that quickly connect as the sphere radius increases and manifest in the barcode as multiple short bars. The topological tortuosity measure proposed for brain arteries (*26*) was based on analyzing data with a simpler tree structure and used information on medium scale bars in the dimension 0 barcode, whereas tumor vessel networks may have multiple components, vary strongly in size, and are more tortuous than brain arteries. To ensure that the tortuosity descriptor did not mistakenly count separate vessels as a single tortuous vessel and was not confounded by network size (i.e., that the descriptor captured topological connectivity), we normalized the descriptor. To enable our descriptor to capture high tortuosity, we focused on short bars in dimension 0 barcodes rather than medium scale bars. Specifically, the tortuosity descriptor proposed here was defined as the ratio of the number of short bars (≤10% of maximal radius used in the radial filtration) in dimension 0 barcodes to the number of vessel segments (see Materials and Methods). Note that considering bars with length of ≤10% of maximal radius as being “short” is a modeling choice. The loop descriptor was computed from the number of bars in dimension 1 barcodes of the radial filtration and divided by the number of vessel segments. We further used the radial filtration to determine how the number of loops per vessel segment changes over time in annuli at different distances from the tumor center. PH of the α-filtration allowed us to identify voids, i.e., avascular tumor regions, and their volume in the vessel networks. Long bars in the corresponding barcodes (dimension 2) represent large voids, while short bars represent small voids. The void descriptor measures the median persistence value or bar length in the barcodes.

### Validation of topological descriptors on two datasets

We validated the topological descriptors on data from studies in which tumors were treated with different agents with known effects on tumor vasculature: vascular targeting agents DC101 and anti-Dll4 in the intravital data and bevacizumab in the ultramicroscopy data (see Fig. 4). We found significant differences in our topological descriptors between control and treatment groups of both datasets despite that (i) the biology in the two studies was different, e.g., treatments, tumor types, and mouse models (see the “Datasets” section in Materials and Methods for description), which can influence the degree of tumor vascularization and blood vessel structure (*53*), and (ii) the imaging modalities are not straightforward to compare (intravital is time course data, can be normalized, and has high spatial resolution in the *x-y* plane but low penetration depth, whereas ultramicroscopy is snapshot data at lower spatial resolution but across the whole tumor). While these technical differences led to discrepancies in computational feasibility and interpretation (see the “Computational differences between datasets” and “Tortuosity in the ultramicroscopy data” sections in the Supplementary Materials), we successfully completed computations and showed that our topological descriptors are interpretable for both datasets (see also the “Alternative results figures and statistical analysis” and “Additional results and statistical analysis” sections in the Supplementary Materials for statistical analysis).

Our tortuosity descriptor and the number of loops per vessel segment succeeded in capturing increased sprouting in the vascular networks induced by anti-Dll4 (see Fig. 4A, i and ii, and figs. S4A, S6, and S7) and confirmed the transient phenomenon of vascular normalization (*3*) induced by DC101 (see Fig. 4A, i and ii). Specifically, the tortuosity descriptor captured vascular normalization 2 days after treatment, in agreement with the literature (*3*), and our loop descriptor showed vessel normalization 2 to 4 days after treatment for loops, which has not been reported before.

**Fig. 5.**
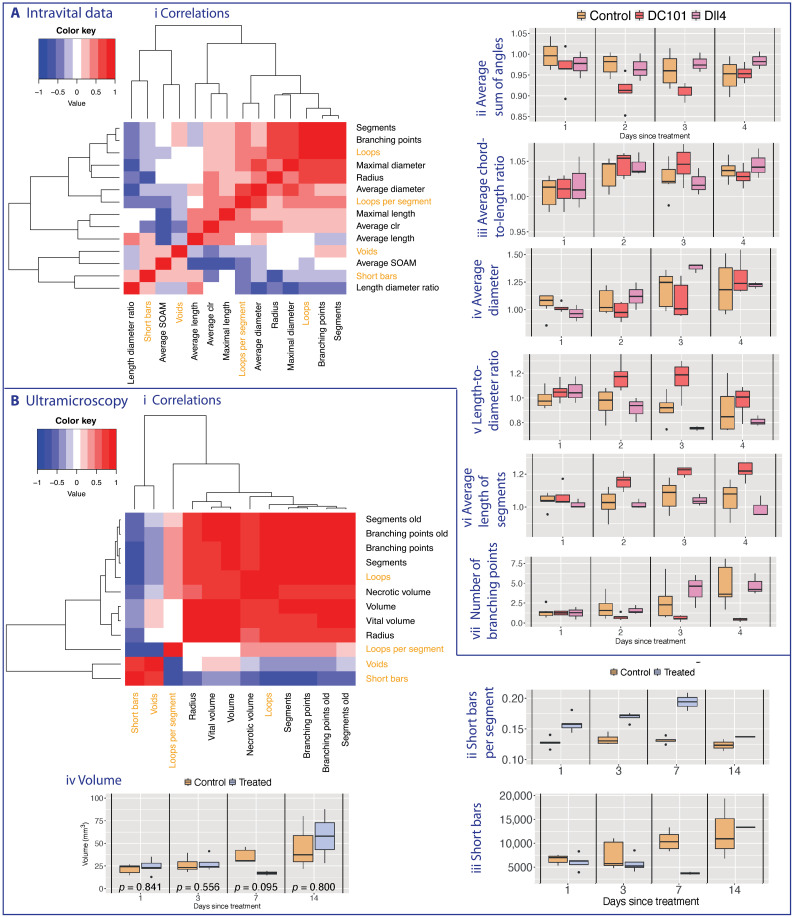
Heatmaps displaying pairwise Pearson correlation coefficients between different vascular descriptors (standard and topological). Vascular descriptors were derived from the (**A**) intravital data and (**B**) ultramicroscopy data. The dendrograms (Ai and Bi) represent complete linkage clustering using the Euclidean distance measure. We compute standard vascular descriptors for comparison (see Section Standard measures and existing descriptors in Introduction and Section Additional results and statistical analysis in SI). We highlight the topological measures in orange including both the number of loops and number of loops per vessel segment to highlight the effect of the normalization. For the ultramicroscopy data, we mark those descriptors that we report from (*8*) with the word “old” (as opposed to the same descriptors that we calculate as in (2,45)). We present existing tortuosity descriptors (Aii and iii) and standard measures on the data (Aiii - vi, Biv). While the proposed topological tortuosity descriptor is a good measure for intravital data (see Fig. 4), care must be taken with the ultramicroscopy data (Bii and iii, see Section Tortuosity in the ultramicroscopy data in SI for details).

For the ultramicroscopy data, care is needed when interpreting the proposed tortuosity descriptor since these networks are less resolved in the *x*-*y* plane, information loss may occur due to computational limitations of filtration step size, and the number of vessel segments reduces markedly following treatment with bevacizumab. These three factors lead to a counterintuitive increase in tortuosity after treatment since small vessels are stepped over by the radial filtration without a finer spatial resolution (either in data or computation). Visual inspection (see Fig. 2) does not show tortuous vessels. If we consider the non-normalized tortuosity descriptor, we observe a decrease in tortuosity compared to control (see Fig. 5Biii and fig. S2). However, as the non-normalized tortuosity descriptor is strongly influenced by network size, it does not capture the desired information (for a more detailed discussion of the tortuosity descriptor in the ultramicroscopy data, see also the “Tortuosity in the ultramicroscopy data” section in the Supplementary Materials).

### Topological loop and void descriptors surpass standard measures

Throughout our analysis, we computed the topological descriptors indexed by a filtration value, which corresponds to tracking the evolution of connectivity at different spatial scales. Therefore, standard (spatially averaged) morphology descriptors are not directly comparable with topological (spatially resolved) descriptors. Performing a comparison required us to compute the topological descriptor for the entire network, losing spatial information encoded in the barcode (see Fig. 4). We report a comparison between standard and topological descriptors and their correlations in Fig. 5. Topological descriptors provided complementary information to standard statistical measures and surpassed them by providing multiscale information of spatial location of tortuosity and connectivity information captured with the loop descriptor (see correlations in Fig. 5A, i, and fig. S23). The tortuosity of the intravital dataset appeared qualitatively consistent with the conventional tortuosity measure mean SOAM across the network (see Fig. 5A, ii, and fig. S15). Our results suggested that the discriminatory power of the tortuosity descriptor for this dataset lies between SOAM and mean clr (see Materials and Methods; Fig. 5A, ii and iii; and figs. S6, S14, and S15). Compared to standard measures calculated on the intravital vascular networks (see Fig. 5A, iv to vii, and figs. S8 to S13), the effect of the treatments on the number of loops highlighted either more significant and discriminatory differences from day 2 after treatment onward (average vessel diameter, maximal vessel diameter, and maximal vessel length) or higher significance on day 3 (number of vessel segments, number of branching points, and average vessel length). In comparison to the length-diameter ratio, the number of loops captured a more prolonged change in network structure that was still discernible on day 4 after treatment.

In the ultramicroscopy data, our loop descriptor further confirmed transient normalization effects of bevacizumab visible 1 to 7 days after treatment (see Fig. 4B, i), whereas the void descriptor captured sustained effects of bevacizumab on angiogenesis (see Fig. 4B, ii). While the topological descriptors showed known effects, these trends could not be explained by changes in standard measures, such as tumor volume (see Fig. 5B and fig. S24) and, therefore, represented genuine structural changes in the degree of vascularization. The avascular regions captured by the void descriptor did not correlate with any existing standard measures (Fig. 5B, i), suggesting that these topological descriptors provide additional quantification of network connectivity. The differences between the treatment groups in the ultramicroscopy dataset were significant for all topological descriptors on days 1 and 3 after treatment (see fig. S4). The void descriptor was ideally suited for this dataset as it contains the full tumor rather than a slice as in the intravital data (see fig. S21).

### Spatiotemporal variation of vascular networks captured by loop descriptor

In contrast to the ultramicroscopy data (see fig. S26), we found spatiotemporal variation in the number of loops in response to different treatments in the intravital data (see Fig. 4A, iii). We divided the radial filtration into different spatial intervals (corresponding to spherical shells around the tumor center) and observed the median number of vessel loops per vessel segment over time in each shell, normalized by day 0 of treatment. We again confirmed known mechanisms of action for vascular targeting agents DC101 and anti-Dll4; anti-Dll4 increased sprouting predominantly from blood vessels close to the tumor periphery, thereby leading to the formation of loops (see orange/red/brown colored sectors in Fig. 4A, iii), whereas DC101 reduced the number of loops across the entire vessel network (see blue colored sectors in Fig. 4A, iii).

### Topological descriptors quantified unknown effects of radiation therapies

**Fig. 6.**
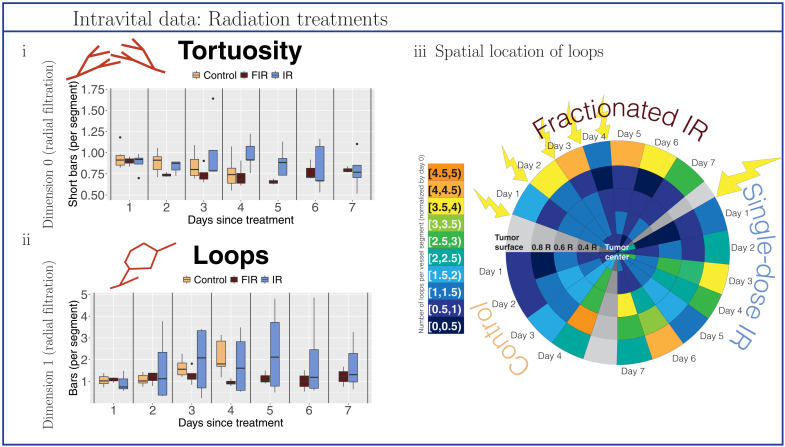
Topological descriptors extracted from tumor blood vessel networks treated with radiation therapy. We normalized all descriptors with respect to values on the day on which treatment is administered (day 0) or, for controls, the day on which observations commenced (day 0). Data were collected from control mice (beige), mice treated with fractionated irradiation (FIR; brown), and mice treated with single-dose irradiation (IR; blue). (**i**) Tortuosity was computed as the ratio of short bars (≤10% of maximal radius used in the radial filtration) in the dimension 0 barcodes of the radial filtration to the number of vessel segments. (**ii**) Loops are the number of bars in the dimension 1 barcodes of the radial filtration per vessel segment in the network. (**iii**) Spatiotemporal resolution of the number of loops per vessel segment. We illustrate the changes in the median number of loops (normalized by day 0) in different radial intervals around the tumor centers over the days of observation. The yellow arrows highlight days for which the tumors have received treatment on the prior day [i.e., an arrow on day 1 signifies that (a dose of) treatment was administered on day 0].

Our topological descriptors quantified and, furthermore, elucidated the unknown effects of single- and fractionated-dose irradiation treatments on vascular networks (see Fig. 6 and figs. S4, S17, and S18). Reductions of tortuosity and the number of loops from single-dose irradiation were apparent only on day 1 after treatment and showed great variation across different tumors over time. Spatially, the effect of single-dose irradiation manifested in a decrease in the number of loops in the whole tumor only on day 1 after treatment and thereafter remained stable only very close to the tumor center; by contrast, the number of loops increased again in most parts of the vessel network (see Fig. 6, iii). Beneficial effects of fractionated-dose irradiation became apparent after a time lag of 2 (tortuosity) or 3 days (loops, with statistically significant difference to controls on day 4; see fig. S4) after initial treatment. Spatially, the number of loops decreased below the tumor surface but increased in the tumor periphery from day 2 after initial treatment onward (see Fig. 6, iii, after the start of fractionated irradiation treatment). Trends in tortuosity and the number of loops revealed changes in network structure and differ from those seen for the approximate tumor radius (see fig. S22).

### Vascular architecture evolution linked to increasing topological complexity

Last, when comparing all five treatment groups in the intravital data, we found significant differences for tortuosity on day 2 after treatment (see fig. S19), followed by significant differences in the number of loops on day 3 after treatment (see fig. S20). We hypothesize that vascular targeting agents and radiotherapy first show effects on the level of tortuosity before changes manifest in more complex network structures such as vessel loops. Biologically, this can be explained by treatments having an immediate effect on individual vessels, while visible changes in network connectivity associated with angiogenesis take longer to occur.

## DISCUSSION

In the present work, we showcased the application of an interpretable and powerful, multiscale topological method to analyze highly resolved images. Our approach represents a much needed paradigm shift in the analysis of images of biological tissues, closing the current gap between the level of detail in data from modern imaging modalities, which are highly resolved over space and time, and coarse quantitative descriptors commonly extracted from these images. We quantified, validated, and uncovered aspects of network connectivity in tumor vasculature by exploiting the three-dimensionality of state-of-the-art data across different scales, from small vessel loops to large voids, information that is inaccessible using standard summaries. Our topological descriptors characterize tortuosity and vessel loops (radial filtration) and tumor vascularization (α-complex filtration) in a novel way, giving unprecedented quantification, in terms of spatial location and connectivity, of dynamic changes in the network architecture of tumor blood vessels during disease progression and treatment. In addition to validating the known dynamic effects of vascular targeting agents on vessel density, we also provided novel quantification of their spatial location effects on the vasculature. Hitherto, we offered a multiscale topological characterization of the effects of radiotherapy on vasculature.

When performing PH, the choice of filtration and its interpretation can reveal different information about a system [see (*33*) for an exploration of different filtrations for applications]. For example, the plane-sweeping filtration is well suited to cases for which there is a clear direction in the data [e.g., brain arteries (*26*)]. Here, we used points sampled from the vessels in 3D space and then constructed the radial filtration and the α-filtration, both of which are robust with respect to rotation. However, the barcode will change with deformation (e.g., loops being stretched) as PH computes information about both geometry and topology. We made the simplifying assumption for the radial filtration that the tumors are spherical, as is often done in modeling; however, this does not hold if the tumor is ellipsoid in shape. For different tumor geometries, the vessels may not be radially oriented, so a future extension could be to consider an ellipsoid filtration (*54*). Similarly, our estimated tumor center is only an approximation of the true tumor center, and results could differ when choosing an alternative starting point, although we expect this difference to be small since the filtration radius increases uniformly throughout the analysis and our spatiotemporal summaries are computed over large intervals. Another possible extension is to consider slightly different points for the tumor center and to generate a collection of persistence diagrams for each tumor. The descriptors extracted from these diagrams would, however, need to be averaged per tumor.

An important aspect of analyzing spatial network data is the robustness of results. PH is stable to noise with respect to perturbation of individual points in the dataset, such as small changes in network edge lengths or small changes in the location of vessel nodes. However, the theoretical PH stability results with respect to bottleneck distance do not hold when entire edges are added or deleted, which may arise due to segmentation or imaging noise. Since we computed the median and standard deviation of topological descriptors, we expect our results to be robust to both sources of noise.

The filtrations we used in this work cannot be applied to all types of tumors; for example, a recent study predicting the survival of glioblastoma motivated a new topological statistic for analyzing shape that effectively analyzes multiple directions of sublevel sets (*55*). However, given the multiscale nature of our topological descriptors, we expect them to scale readily to human tumors, given a level of imaging detail similar to that used here. It should be possible to use photoacoustic imaging (*56*, *57*) to construct accurate vascular network outlines in experimental settings in living human subjects with superficial cancers (e.g., breast and skin cancers). For such images of human tumors, we would use the same pipeline for segmentation, TDA computations, and to generate descriptors. For larger networks, we may use the subsampling step before TDA analysis, as we did here for the ultramicroscopy data.

We found that the utility of different topological descriptors (i.e., dimensions) may depend on imaging resolution. Our results indicated that the high planar resolution of intravital data better captures tortuosity, resulting in more short connected components in the radial filtration, whereas it is less appropriate for measuring voids because of the shallow imaging depth. Conversely, the deep *z* direction of ultramicrosopy enables quantification of voids (i.e., higher-dimensional homology features), while comparably low planar resolution may not suffice to generate the small features in dimension 0 needed to quantify tortuosity. We demonstrated that loops were well quantified for both modalities. We specifically chose descriptors that are simple summaries of PH barcodes so that we can interpret biological differences between networks. In other biological applications, barcodes have been successfully analyzed by their vectorization, for example, by using persistence images (*58*) or persistence landscapes (*59*, *60*) and classifying them using methods from machine learning; see, for example, (*22*, *36*, *61*–*64*). Here, this type of transformation is not suitable because of the variation of initial vasculature within a treatment group and small sample size common to mouse model experiments. In the future, we will investigate more computationally efficient and interpretable topological invariants, such as the Euler characteristic and Betti numbers, which can be computed directly via the Euler characteristic rather than via a filtration and also provide quantitative information about vessel connectivity. However, the simplified computation will come with a loss of information; for example, we will not be able to compute our tortuosity descriptor or the size of voids. In other future work, we will extract the spatial locations of the tumor and immune cells from the images and apply similar topological analyses to these data to compare control and treated tumors.

Our topological descriptors provide global and multiscale quantification of vascular connectivity and represent a first step toward understanding the relationship between the structure and function of the vasculature. For example, high tortuosity of vessels has been observed to reduce blood flow (*65*), and a future extension will be to develop directed topology approaches for tackling such directed vascular networks. Even with the state-of-the-art data used here, ethical constraints preclude the collection of more data, thereby limiting the strength of the biological conclusions that can be drawn. If more data were available, our topological descriptors could be fed directly into machine learning algorithms and analyses. Since our datasets were obtained from two different studies, we were unable to directly compare them. The data were generated on different scales, and therefore, the values differ substantially, with respect to regions of interest and spatial resolution. In future work, it would be informative to cross-validate the descriptors by performing intravital imaging followed by ultramicroscopy imaging on the same mice with small tumors to validate the method on the same vessel networks, exclude any influences from different imaging modalities, and work toward topological data integration. At this time, analysis of the same tissue with both imaging modalities is logistically impossible. We propose that the topological descriptors be tested with different imaging modalities used in the clinic to determine their practical use for monitoring the response of tumors to therapy.

We conclude by noting that the topological perspective for analyzing and preserving the multiscale nature of data is broadly applicable to other spatial networks (*32*) and biological systems, where it can also be used to quantify perturbations to network topology. Such networks not only arise across many different biomedical applications but are also relevant in other biological settings ranging from leaf vessel networks to collagen fibers and signaling networks.

## MATERIALS AND METHODS

### Experimental procedures for intravital data

#### Abdominal imaging window implantation

This procedure was based on a previously described method (*6*). Mice were prepared in a surgical unit, administered with inhalational anesthesia and preoperative analgesics. Body temperature and respiration rates were monitored throughout the procedure. A 1-cm cut was made along the abdominal midline approximately 5 mm underneath the sternum followed by blunt dissection around the cut to separate the connective tissue from the skin. A custom-made imaging window frame (Workshop at the Department of Oncology, Oxford University) was fitted underneath the skin. Continuous sutures were used to secure the skin to the window frame. Approximately 2.5 × 10^5^ MC38 cells stably expressing enhanced green fluorescent protein (eGFP) in 5 μl containing 30% of Matrigel and 10% of Evan’s blue dye were injected under the connective tissue and above the abdominal muscle layer. The chamber was then flushed with water to lyse noninjected cells by osmotic shock, tapped dry with sterile cotton swabs, and flooded with saline. A cover glass glued on the chamber’s lid was secured onto the window frame. The animals were then placed onto a heat mat for postoperative recovery, and their health and tumor growth were monitored by visual examination.

#### Treatment regimes

Animals with tumors approximately 100 mm^3^ in the chamber were administered with either anti-mouse vascular endothelial growth factor receptor 2 (VEGFR2) antibody [27 mg/kg; clone DC101 (*37*), BioXCell], anti-mouse Dll4 antibody (*39*) twice per week at a dose of 5 mg/kg (in two doses on the initial day of imaging and 3 days later), or one of two radiation treatments. For the radiation treatments, mice were anesthetized under inhalation with isoflurane and placed in an imaging-guided small animal radiation research platform (SARRP) irradiator (Xstrahl Ltd). A Cone Beam CT (computerized tomography) scan of each mouse was obtained, and the treatment was planned using MuriPlan (Xstrahl Ltd). The SARRP was used to deliver 15 Gy of x-rays (220–kilovolt peak copper-filtered beam with half-value layer of 0.93 mmCu) to the tumor at 2 Gy/min. This was given either in a single dose or at five daily fractionations of 3 Gy of x-ray radiation to the tumor. Dosimetry of the irradiator was performed as previously described (*66*).

#### Intravital two–photon imaging

To visualize the tumor vasculature, we used a transgenic mouse model in which the fluorescent protein tdTomato is expressed in both normal and tumor endothelial cells (TECs). We used transgenic mice bearing a Cre recombinase–tamoxifen receptor fusion protein (Cre-ERT2) driven by the VE cadherin promoter. These mice were crossed with Gt(ROSA)26Sortm9(CAG-tdTomato)Hze mice so that activation of Cre by tamoxifen resulted in EC expression of tdTomato (schematic shown in fig. S1). Similarly, TECs, identified as CD31-positive cells in allografted tumors, were rendered generally over 90% tdTomato positive (fig. S1). For imaging purposes, we only used mice with greater than 95% fluorescent EC.

Mice were imaged for 4 days following initial treatment for vascular targeting agents and 7 days for radiation treatment with a Zeiss LSM 880 microscope equipped with an aesthetic vaporizer and respiratory monitoring system. Stage and atmosphere were heated to 37°C. To label perfused vessels, Quantum dot-705 solution (1 μM; Invitrogen) was infused intravenously using a motorized pump at a rate of 0.84 μl/min. A mode-locked Mai Tai laser tuned to 920 nm was used to simultaneously excite eGFP, tdTomato, and Qdot705. The Qdot705 signal was acquired through a BP700/100 filter with a nondescanned detector. Gallium arsenide phosphide detectors were used to acquire the signal of tdTomato selected by a BP650/45 filter and the eGFP selected by a BP525/50 filter. Images were acquired in *z*-stack tile scans with a pixel size of 0.823 μm and an image size per tile of 512 × 512 × 5 in *x*, *y*, and *z*, respectively. A water immersion 20× objective made for ultraviolet-visible–infrared transmission with a numerical aperture of 1 was used. The segmentation of tumor blood vessels was based on the TECs expressing tdTomato. We used intravenous injection of Qdots to distinguish perfused from nonperfused tumor vessels, i.e., vessels labeled with the infused Qdots and vessels not labeled with it. As further evidence, we note that no Qdot-positive, endothelial-negative vessels were identified. If the Qdots were in the lymphatics, then they would have identified vessels not lined by vascular endothelium; this did not happen. All animal experiments were conducted in accordance with the U.K. Animals (Scientific Procedures) Act 1986 as amended [Amendment Regulations 2012 (SI 2012/3039)], under the authority of a U.K. Home Office Project Licence (PPL 30/2922 and PCDCAFDE0), with local ethical approval from the University of Oxford Animal Welfare and Ethical Review Panel.

### Datasets

We analyzed two different tumor blood vessel datasets: data obtained by multiphoton intravital 3D imaging (*5*) (see above for description of experimental procedures) and data obtained by ultramicroscopy (*7*). Both datasets consist of 3D stacks of images of tumor blood vessels subjected to different experimental conditions.

#### Dataset I: Multiphoton intravital 3D imaging

The intravital dataset consists of tumor vasculature images that were obtained from multiphoton intravital 3D imaging (*5*) of transgenic mice injected with murine colon adenocarcinoma cells (cell line MC38). The animals were imaged alive and over several days using the experimental procedures described in the “Experimental procedures for intravital data” section. The mice were divided into groups that were subjected to different experimental conditions:

1) Controls (seven mice).

2) Anti-Dll4–treated tumors (three mice): The mice were treated using anti-Dll4 antibodies (*39*), which block Dll4 signaling and thereby increase vessel sprouting. The resulting networks are very dense and complex.

3) DC101-treated tumors (five mice): The mice were treated using DC101 antibodies (*37*), which block VEGFR2 signaling and thereby reduce vessel sprouting.

4) Single-dose irradiated tumors (five mice): The mice were treated with a single dose of 15 Gy on the first day of imaging.

5) Dose-fractionated irradiated tumors (four mice): The mice were treated with five doses of 3 Gy over five consecutive days followed by 2 days of rest starting on the first day of imaging.

In each case, we refer to the start of treatment or observation as day 0.

#### Dataset II: Multispectral fluorescence ultramicroscopy data

The ultramicroscopy dataset consists of multispectral fluorescence ultramicroscopy (*7*) images of blood vessels of human breast cancer tumors [cell line KPL-4, human epidermal growth factor receptor 2 (HER2) positive] that were implanted into 31 immunodeficient mice. The experiments were carried out by Dobosz *et al.* (*8*), Roche Diagnostics/Institute for Biological and Medical Imaging, Helmholz Zentrum, Munich. The mice were divided into a control group and a treatment group:

1) Controls (18 mice).

2) Anti–VEGF-A–treated tumors (13 mice): The mice were treated with bevacizumab (*43*), an antibody that binds to VEGF-A and thereby induces normalization (*3*) of the vessel networks, i.e., reduces some of their structural and functional abnormalities and lowers their permeability (*8*).

Treatment was administered once the tumors reached a volume of approximately 60 mm^3^, and controls were observed accordingly. To test the effect of treatment on drug delivery at different time points, both controls and anti–VEGF-A–treated mice were also treated with trastuzumab (*67*) (anti-HER2 antibody) 6 hours before the tumor was extracted and prepared for imaging. Different subgroups of tumors were imaged on day 1 (five controls and five treated), day 3 (five controls and four treated), day 7 (five controls and two treated), and day 14 (three controls and two treated) after administration of bevacizumab. For more details on experimental conditions, see (*8*) [note that the dataset in (*8*) was created under the same conditions and overlaps with the data used in this work, but the two are not identical, e.g., the dataset in (*8*) consists of five controls and treated mice for days 1, 3, and 7 after treatment each but does not include day 14 after treatment]. Imaging was performed ex vivo at a spatial resolution of 5.1 μm on the *x-y* plane with images taken every 5.1 μm in the *z* direction. Skeletonizations of the images were produced by Dobosz *et al.* (*8*) using a custom Definiens Developer script. For details of how the ultramicroscopy data were skeletonized, please see the “Data preprocessing” section in Materials and Methods.

### Data preprocessing

#### Intravital data

Skeleton files were extracted from the imaging data by combining two segmentation models and taking their geometric average. The skeletons were then pruned [see (*45*), p. 165, for a full description]. The segmentation method used for the intravital dataset was extensively tested against synthetic datasets and against manually segmented intravital microscopy images (*45*). This method achieved a Dice score of 0.97. Moreover, a skeleton error (given in micrometers), the distance between skeletons that was computed using the modified Hausdorff distance, was determined. This skeleton error can be interpreted as the average shortest distance between any point on the ground truth skeleton and some point on the target skeleton and vice versa. With our method, this skeleton error was 5 μm compared to ground truth in the synthetic dataset and in the intravital dataset, where manually segmented images were considered as ground truth. Last, the method used also achieved coverage of 0.96 to 0.99 both in the synthetic datasets and intravital microscopy datasets. This shows that the errors introduced by the segmentation method were relatively small.

We extracted blood vessel networks from skeleton files using the method VesselTree from unet_core.vessel_analysis in the python code package unet-core (*44*). The extracted networks consist of points on vessel branches (multiple points per vessel branch including branching points), which represent the network nodes, and the vessels that connect them, which constitute the edges of the network. VesselTree also enables us to extract network features such as the number of vessel segments (i.e., edges of the network), number of branching points (i.e., nodes of the network), vessel diameters, vessel lengths, and measures of tortuosity (clr and SOAM) for every point. We account for the difference in resolution between the *z* axis and the *x-y* plane by rescaling the coordinates in the *z* direction using a factor of $\frac{0.83}{5}$ on the *z* coordinates before further analysis.

We excluded the following data from our analysis because of imaging and/or segmentation quality: control tumor 24_2C, day 4; fractionated-dose irradiated tumor 60_1E, day 5 onward. For the radial filtration, because of the very high number of points in some of the blood vessel networks, we reduced the data size by including all branching points but sampling only every second point from every branch in the following networks: control tumor 18_4E, day 3; control tumor 18_4E, day 4; control tumor 29_1B, day 3; control tumor 29_1B, day 4; control tumor 34_2A, day 4; control tumor 60_2A, day 4; DC101-treated tumor 51_2C, day 1; DC101-treated tumor 54_2D, day 2; anti-Dll4–treated tumor 24_2A, day 3; anti-Dll4–treated tumor 24_2A, day 4. The days listed refer to the days after tumor treatment. For the α-complex filtration, we used the full set of nodes as input.

#### Ultramicroscopy data

We preprocessed grayscale skeletonization files provided in the ultramicroscopy dataset from individual .tif files (one for every *x-y* plane slice of the vessel network) to .tif stacks in uint8 format using the software ImageJ (*68*). We then converted the .tif stacks to .nii format using the function tiff2nii.m from the Matlab toolbox (*69*). We used the .nii files as input for our unet-core (*44*) in our python scripts. Although unet-core was originally trained on multiphoton intravital 3D imaging, we justify our approach by the fact that the skeletonizations are clear, high-contrast images (see Fig. 2 for extracted networks). Any imaging-specific effects were removed by the skeletonization process that was developed specifically for this dataset (*8*). We compared the number of branching points and the number of vessel segments extracted by unet-core with similar measurements extracted previously by Dobosz *et al.* (*8*) and found that these are highly correlated (see Fig. 5B and fig. S28).

We note that we obtained 3D coordinates for network nodes. The distances between these nodes scale linearly with the true distance in micrometers. Since we were only interested in features with respect to their relative distance to the tumor center, this was sufficient for our purposes. A coordinate set true to distance could be obtained by comparing an exemplary output network closely to microscopy images.

For the radial filtration, because of the very high number of points in these blood vessel networks (on the order of millions of nodes in comparison with on the order of thousands of nodes in the intravital data), we reduced the point clouds for all tumors by including all branching points but sampling only every fourth point from every branch. Despite our reduction approaches, we were not able to run our codes on one of the treated tumors from day 14 networks. For the α-complex filtration, we used the full set of nodes as input.

### Topological data analysis

TDA is an umbrella term used for methods that allow the study of potentially high-dimensional data using mathematical concepts from topology (*70*). PH (*16*–*19*) quantifies global topological structures (e.g., connectedness, loops, and voids) in data. More details on TDA and PH are in the Supplementary Materials.

#### Homology and simplicial complexes

To compute (persistent) homology from data, we first constructed simplicial complexes, which can be thought of as collections of generalized triangles. From the constructed simplicial complexes, we quantified and visualized the datasets’ connected components (dimension 0), loops (dimension 1), and voids (dimension 2) at different spatial scales in the data.

PH is based on the topological concept of homology [for intuitive introductions, see, for example, (*23*, *71*); for more formal introductions, see (*72*–*74*)]. To compute topological invariants, such as connected components (dimension 0) and loops (dimension 1), we used homology. To obtain homology from a simplicial complex, *X*, we constructed vector spaces whose bases are the 0-simplices, 1-simplices, and 2-simplices, respectively, of *X*. There is a linear map between 2-simplices and 1-simplices called the boundary map ∂_2_, which sends triangles to the edges on their boundary. Similarly, the boundary map ∂_1_ sends edges to their boundary vertices (or boundary points), and ∂_0_ sends vertices to 0. The action of the boundary map ∂_1_ on the simplices is stored in a binary matrix where the entry *a*_*i*, *j*_ denotes whether the *i*th 0-simplex forms part of the boundary of the *j*th 1-simplex. If so, then *a*_*i*, *j*_ = 1; otherwise, *a*_*i*, *j*_ = 0. We computed the kernel Ker( · ) and image Im( · ) of the boundary maps to obtain the vector spaces *H*_0_(*X*) = Ker (∂_0_)/ Im (∂_1_) and *H*_1_(*X*) = Ker (∂_1_)/ Im (∂_2_). Note that for the radial filtration, we have Im(∂_2_) = 0 since we do not fill in any triangles in the filtration but only have edges and vertices. These vector spaces are also referred to as homology groups, and their dimensions define the topological invariant that we studied called the Betti numbers of *X*, β_0_ and β_1_, which give the number of connected components and loops, respectively. We studied a vascular network at multiple scales in different ways as will be described later in this section. The multiple scales of the data can be encoded by a filtration, which is a sequence of embedded simplicial complexes *X*_0_ ⊆ *X*_1_… ⊆ *X*_end_ built from the data.

#### Persistent homology

PH is an algorithm that takes in data via a filtration and outputs a topological summary, which visualizes changes in topological features such as connectedness (dimension 0) and loops (dimension 1) across the filtration. The simplicial complexes are indexed by the scale parameter of the filtration. The inclusion of a simplicial complex *X_i_* ⊆ *X_j_* for *i* ≤ *j* gives a relationship between the corresponding homology groups *H_p_*(*X_i_*) and *H_p_*(*X_j_*) for *p* = 0,1,2. This relationship allowed us to track topological features such as loops along the simplicial complexes in the filtration. Intuitively, a topological feature is born in filtration step *b* when it is first computed as part of the homology group *H_p_*(*K_b_*) and dies in filtration step *d* when that feature no longer exists in *H_p_*(*K_d_*), i.e., when a connected component merges with another component or when a loop is covered by 2- simplices. The output from PH is a multiset of intervals [*b*, *d*) that quantifies the persistence of topological features. Each topological feature is said to persist for the scale *d* − *b* in the filtration.

#### Method I: Radial filtration

**Fig. 7.**
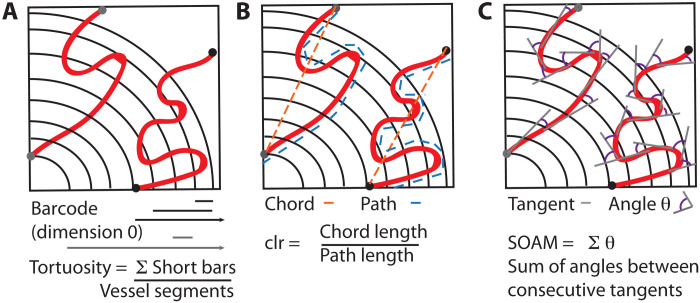
Schematic of tortuosity descriptors. (**A**) The topological descriptor is defined as the number of short bars in the barcode (connected components in dimension 0 barcodes with persistence of ≤10% of the maximal radius) normalized by the number of vessel segments. In this schematic, there are two vessels. This normalization ensures that the connected components in the tortuosity measure do not also count different vessel segments. This descriptor is in contrast to the topological tortuosity reported in (*26*), which did not have multiple vessel segments. (**B**) The clr (*45*) is the ratio between the chord connecting two ends of a curve (orange) and the path length of the curve (blue). Clr measures the deviation from a straight line. (**C**) The SOAM measures the sum of angles between consecutive tangents of a curve, so a high score is given to a curve rapidly changing direction.

We applied a radial filtration (*33*) to the 3D vessel networks, i.e., the collection of nodes (both branching points and points along vessel branches), their spatial coordinates, and the edges between them. We built the filtration starting in the tumor center, which we approximated by the center of mass of the points sampled from the tumor blood vessels, e.g., the nodes of our networks. We then proceeded in the following way. We divided the maximal distance of a node in the network to the center of mass into 500 steps and, from this, constructed a sequence of uniformly increasing radii. By increasing the radial distance stepwise, in each filtration step, we included all nodes within the specified radius. If two nodes that were connected by an edge were also within the given radius, then we added that edge to our filtration. In the barcodes from this filtration, we could capture tortuosity (from connected components in dimension 0 barcodes with persistence of ≤10% of the maximal radius used; see Fig. 7A), loops (dimension 1), and their spatial distribution. We note that for the intravital (shallow) imaging data, the approximated tumor center is defined by the image (vessel nodes) viewed through the window chamber. The approximated tumor center was calculated on the basis of the vasculature in this small segment and hence does not represent the true tumor center.

#### Method II: α-Complex

On 3D data, the α-complex (*20*, *75*) filtration builds a sequence of nested simplicial complexes (collections of nodes, edges, triangles, and tetrahedra) whose final element *K*_end_ is the Delaunay triangulation (*48*), i.e., the triangulation of the 3D convex hull of the data points by tetrahedra. We built the filtration on the 3D nodes of the vessel networks. Inductively, starting with the highest dimension (i.e., first tetradedron, then edges), each simplex σ in *K*_end_ was assigned a filtration value given by the square of its circumradius α in the case that the circumsphere contains no other vertices than the vertices of σ; otherwise, its filtrations value was given by the minimum of the filtration values of the higher-dimensional simplices of which σ was a face. To construct the filtration, edges, triangles, and tetrahedra were included up to a set filtration value that increased stepwise. The effect of the assignment of the filtration values was, for example, that, in 2D, the long edge of a slim triangle was only included when the whole triangle was included. This avoided the formation of cycles for slim triangles. In the barcodes from this filtration, we could capture the degree of tumor vascularization (from voids, dimension 2).

### Existing descriptors

The standard morphological descriptors that we computed from the segmented intravital microscopy images were the number of vessel segments (i.e., number of edges), number of branching points (i.e., number of nodes), maximal vessel diameter, average vessel diameter, maximal vessel length, average vessel length, average clr, average SOAM, and vessel length/diameter ratio. The standard descriptors that we included for the ultramicroscopy dataset were the number of vessel segments [both as computed in (*8*) and unet-core (*44*)], number of branching points [both as computed in (*8*) and unet-core (*44*)], necrotic tumor volume as computed in (*8*), tumor volume as computed in (*8*), and vital tumor volume as computed in (*8*).

#### Tortuosity: SOAM

The SOAM was applied as a measure of tortuosity in blood vessels by Bullitt *et al.* (*13*). It is the sum of the angles of regularly sampled tangents along a blood vessel skeleton and can take values from zero (straight vessel) to infinity. For tortuous vessels, the metric increases monotonically with vessel length. See Fig. 7B for a schematic.

#### Tortuosity: clr

The clr (*45*) of a blood vessel is defined as the ratio of the distance between the branching/end points of the vessel and the length of the vessel. The measure can take a value of, at most, one (straight vessel) and tends to be zero for very tortuous vessels. See Fig. 7C for a schematic.

#### Statistical analysis

We analyzed the statistical significance of differences between treatment groups in the tortuosity values, number of loops per vessel segment, and median persistence of voids. We performed a pairwise Wilcoxon rank sum test on the ultramicroscopy data for each day separately to determine the statistical significance of our topological measures (see fig. S4B). We tested at a significance level of 0.05. For the intravital data, we performed a Kruskal-Wallis test to determine whether at least one treatment group differs significantly from the others for the topological descriptors (see figs. S6, S7, and S17 to S21) and for standard vasculature measures (see figs. S6 to S16). We further applied a pairwise Wilcoxon rank sum test between the control group and the different treatment regimes for the topological descriptors (see fig. S4A). We again tested at a significance level of 0.05 and did not correct for false discovery rate. To explore correlations between different types of summary descriptors for vascular networks, we computed pairwise Pearson correlation values for the different descriptors in both datasets separately (see figs. S23 and S28). We performed all statistical analyses in R Studio (*76*), and all our tests described above were, by default, two-sided.

### Implementation

We implemented the radial filtration in Matlab and used the software package javaPlex (*77*) (note that javaPlex was last updated in July 2018; alternatively, the well-maintained GUDHI library (*78*) also allows the user to build their own simplicial complex and/or filtration on data using the class SimplexTree) (*77*) to compute PH on our filtration. We divided the distance from the tumor center (center of mass) to the farthest away point in the blood vessel network into 500 steps to build the radial filtration. We implemented the α-complex using the GUDHI library (*78*). All code is freely available at the following repository: https://github.com/stolzbernadette/TDA-Tumour-Vasculature.

## Supplementary Materials

### This PDF file includes:

Topological data analysis Computational differences between datasets Supporting experimental information Tortuosity in the ultramicroscopy data Alternative results figures and statistical analysis Additional results and statistical analysis Table S1 Figs. S1 to S28 References

## Notes

View/request a protocol for this paper from *Bio-protocol*.
