# Supplementary material for: Multiscale topology characterizes dynamic tumor vascular networks
Source: Sci Adv. 2022 Jun 10;8(23):eabm2456. doi: 10.1126/sciadv.abm2456 (PMC9187234; doi:10.1126/sciadv.abm2456)
Supplement: Supplementary file 1 — Topological data analysis Computational differences between datasets Supporting experimental information Tortuosity in the ultramicroscopy data Alternative results figures and statistical analysis Additional results and statistical analysis Table S1 Figs. S1 to S28 References [file sciadv.abm2456_sm.pdf]

Science Advances
AAAS

Supplementary Materials for

Multiscale topology characterizes dynamic tumor vascular networks

Bernadette J. Stolz *et al.*

Corresponding author: Bernadette J. Stolz, stolz@maths.ox.ac.uk;
Heather A. Harrington, harrington@maths.ox.ac.uk

Sci. Adv. **8**, eabm2456 (2022)
DOI: 10.1126/sciadv.abm2456

This PDF file includes:

Topological data analysis
Computational differences between datasets
Supporting experimental information
Tortuosity in the ultramicroscopy data
Alternative results figures and statistical analysis
Additional results and statistical analysis
Table S1
Figs. S1 to S28
References

Topological data analysis

Homology and simplicial complexes. Persistent homology is based on the topological concept of *homology* (for intuitive introductions, see, for example, references (*23, 71*); for more formal introductions see references (*72–74*)). Homology allows one to study shapes and forms disregarding any changes caused by stretching or bending. One can study the properties of a topological space by partitioning it into smaller, topologically simpler pieces, which when reassembled include the same aggregate topological information as the original space. Topological spaces can be very simple. Two trivial examples are the empty set $X = \emptyset$ or a space that consists of one single point $X = \{x\}$. If we want to capture the topological properties of the second example $X = \{x\}$, we could simply choose a single node to represent it. However, a node or even a collection of nodes does not allow one to capture the topological properties of more complicated spaces, such as a 2-sphere or the surface of the earth. In such cases, one needs a simple object that carries the information that the space is connected but also encloses a hole. Consider, for example, a collection of triangles glued together to form a hollow tetrahedron; this is an example of a mathematical object called a *simplicial complex*. The building blocks that one uses to approximate topological spaces are called n-*simplices* which one can think of as generalised triangles. The parameter n indicates the dimension of the simplex. Every n-simplex contains $n+1$ independent nodes: a point is a 0-simplex, an edge is a 1-simplex, a triangle 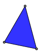 is a 2-simplex, and a (solid) tetrahedron 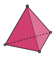 is a 3-simplex. By using a numbering x_i of vertices, we can write a 0-simplex as $[x_0]$, a 1-simplex as $[x_0, x_1]$, a 2-simplex as $[x_0, x_1, x_2]$, and a 3-simplex as $[x_0, x_1, x_2, x_3]$. The lower-dimensional simplices form so-called *faces* of the associated higher-dimensional objects. One combines different simplices into a *simplicial complex* X to capture all different aspects of a topological space. Two simplices that are part of a simplicial complex are allowed to intersect only in common faces. The *dimension* of a simplicial complex is defined to be the dimension of its highest-dimensional simplex. A subcollection

of a simplicial complex X is called a *subcomplex* of X if it forms a simplicial complex itself.

For every simplicial complex X we can define a vector space $C_n(X)$ that is spanned by its n-simplices with coefficients in the field $\mathbb{Z}/2\mathbb{Z}$. The elements of the vector space $C_n(X)$ are called *n-chains*. We can now define a linear map, the so-called *boundary operator*, between vector spaces $C_n(X)$ and $C_{n-1}(X)$ which takes every n-simplex x to the (alternating) sum of its faces, i.e. its boundary:

$$\partial_n : C_n(X) \to C_{n-1}(X),$$
$$x \mapsto \sum_{j=0}^{n} (-1)^j [x_0, \ldots, x_{j-1}, x_{j+1}, \ldots, x_n], \tag{1}$$

i.e. in the j-th summand we omit the vertex x_j from the vertices spanning the $(n-1)$-simplex. Note that the sum in Equation (1) is over the field $\mathbb{Z}/2\mathbb{Z}$ where $(-1) = 1$, i.e. we can omit the $(-1)^j$ term in the above equation. We can use the boundary operator to connect all n-chains of a simplicial complex X in a sequence, the so-called *chain complex* $\mathcal{C} = \{C_n, \partial_n\}$:

$$\ldots \xrightarrow{\partial_{n+2}} C_{n+1} \xrightarrow{\partial_{n+1}} C_n \xrightarrow{\partial_n} C_{n-1} \xrightarrow{\partial_{n-1}} \ldots \xrightarrow{\partial_1} C_0 \tag{2}$$

$$c \longmapsto \partial_n c. \tag{3}$$

We can represent a collection of edges that are connected to form a loop in a simplicial complex as a 1-chain, for example, $[x_0, x_1] + [x_1, x_2] + \cdots + [x_j, x_0]$. If we apply the boundary operator to this 1-chain, we obtain $\partial([x_0, x_1] + [x_1, x_2] + \cdots + [x_j, x_0]) = [x_1] - [x_0] + [x_2] - [x_1] + \cdots + [x_0] - [x_j] = 0$. In contrast, for a collection of edges that does not form a loop this is not the case, e.g., $\partial([x_0, x_1] + [x_1, x_2] + \cdots + [x_{j-1}, x_j]) = [x_j] - [x_0] = [x_0] + [x_j]$ (for coefficients from $\mathbb{Z}/2\mathbb{Z}$). Chains that are in the kernel of ∂_n, i.e. their boundary is zero, are called *n-cycles*. One can compute that the composition of two boundary maps yields zero, i.e.

$$\partial_n \partial_{n+1} c = 0, \tag{4}$$

since the boundary of a boundary is empty. The image $\operatorname{im} \partial_{n+1}$ of the boundary operator is therefore a subspace of the kernel $\ker \partial_n$ and its elements are called *n-boundaries*.

One can associate a family of vector spaces known as *homology groups* to a simplicial complex X based on its cycles and boundaries. For every dimension $n \geq 0$ one defines the nth *homology group* as:

$$H_n(X) = \frac{\ker \partial_n}{\operatorname{im} \partial_{n+1}}. \tag{5}$$

In dimension 2, the elements of the homology group H_2 are called *voids*; in dimension 1, the elements of the homology group H_1 are called *loops*; in dimension 0, the elements of the homology group H_0 are called *connected components*. Two elements in H_n are considered to be different, if they differ by more than a boundary, i.e. if they represent different n-dimensional holes. We then say that they belong to different *homology classes*.

We measure the number of n-dimensional holes of a simplicial complex by considering its nth *Betti number* β_n:

$$\beta_n = \dim H_n(X) = \dim \ker \partial_n - \dim \operatorname{im} \partial_{n+1}. \tag{6}$$

The first three Betti numbers, β_0, β_1, and β_2, represent, respectively, the number of connected components, the number of 1-dimensional holes, and the number of 2-dimensional holes (i.e. voids) in a simplicial complex.

Persistent homology. While homology gives information about a single simplicial complex, PH allows one to study topological features across embedded sequences, so-called *filtrations*, of simplicial complexes, which can be constructed from data. A filtration (*17, 18, 49*) of a simplicial complex X is a sequence of embedded simplicial complexes,

$$\emptyset = X_0 \subseteq X_1 \subseteq X_2 \subseteq \cdots \subseteq X_{end} = X\ , \tag{7}$$

starting with the empty complex and ending with the entire simplicial complex X. The simplicial complexes in the filtration are connected by inclusion maps. One can now apply an important property of homology, *functoriality*: any map between simplicial complexes $f_{i,j} : X_i \to X_j$ induces a map between their n-chains $\tilde{f}^n_{i,j} : C_n(X_i) \to C_n(X_j)$ which induces a map between their homology groups $f^n_{i,j} : H_n(X_i) \to H_n(X_j)$. In particular, this means that there exist maps between the homology groups of every simplicial complex in a filtration, e.g., there are maps that relate the voids, loops or connected components in simplicial complexes across a filtration. One can visualise topological features such as loops or connected components across a filtration in a summary diagram called a *barcode* (*49, 79*). For an appropriate choice of basis (*80*) of the homology groups H_n, a barcode represents the information carried by the homology groups and the maps $f^n_{i,j} : H_n(X_i) \to H_n(X_j)$. A topological feature of dimension n in $H_n(X_b)$ is *born* in $H_n(X_b)$, if it is not in the image of $f^n_{b-1,b}$. For example, intuitively, a loop is born in filtration step b, if the loop appears closed in the simplicial complex X_b for the first time. A topological feature from $H_n(X_i)$ *dies* in $H_n(X_d)$, where $i < d$, if d is the smallest index such that the feature mapped to zero by $f^n_{i,d}$. If the topological feature is a loop, intuitively it dies in the filtration step where it is first fully covered by triangles (or other higher-dimensional simplices). Note that some topological features never die in a filtration, for example, we always have one connected component in a non-empty simplicial complex that is never mapped to zero. In a barcode, topological features in the filtration of a simplicial complex are represented by half-open intervals $[b, d)$. The lifetime of a topological feature, the so-called *persistence* p, is defined as $p = d - b$. For topological features that persist until the last filtration step (and beyond), the persistence is said to be infinite.

Data set	Branching points	Segments (edges)	Tumour volume (initial day of imaging)	Penetration depth	Radial filtration computation per network
Intravital	240 – 10 025	260 – 10 060	100 mm^3	300 μm	Days to weeks
Ultramicroscopy	12 500 – 118 000	16 700 – 169 150	60 mm^3	$\geq$ 5 mm	Weeks to months

Table S1: Summary of data sets, experimental conditions, and computational time.

Computational differences between datasets

The differences in the biology and the imaging of our datasets led to discrepancy in computational feasibility (see Table S1). In particular, the network sizes and penetration depth of the imaging differed considerably, which significantly affected the computations for the radial filtration. We first performed the majority of computations for the intravital data on a IBM System x3550 M4 16 core server with 768 GB RAM over 3 months but were not able to obtain all results. For the ultramicroscopy data as well as the remaining intravital data, we required a Dual Intel Xeon Gold 6240M 18 core processor system with 3TB of RAM to complete computations over further 3 months. While for the intravital data we were able to compute the radial filtration on all networks in the dataset (in some cases after reduction approaches for the number of nodes, see Data preprocessing in Materials and Methods), in the ultramicroscopy data, we were not able to obtain results for one of the control tumours on day 14 of observation despite reducing the number of nodes (see Section Data preprocessing in Materials and Methods).

Supporting Experimental Information

In order to visualize the response of the tumor vasculature, we used a transgenic mouse model in which the fluorescent protein tdTomato is expressed in both normal and tumor endothelial cells (EC). We used transgenic mice bearing a Cre recombinase-tamoxifen receptor fusion protein (Cre-ERT2) driven by the VE cadherin promoter. These mice were crossed with Gt(ROSA)26Sortm9(CAG-tdTomato)Hze mice so that activation of Cre by tamoxifen resulted in EC expression of tdTomato (schematic shown in Figure S1A). For imaging purposes we only used mice with greater than 95% fluorescent EC. The segmentation of tumor blood vessels was based on the TECs expressing tdTomato. We used intravenous injection of Qdots 705 to distinguish perfused from non-perfused tumor vessels, i.e. vessels labelled with the infused Qdots and vessels not labelled with it. As further evidence, we note that no Qdot positive, endothelial negative vessels were identified. If the Qdots were in the lymphatics then they would have identified vessels not lined by vascular endothelium; this did not happen.

Tortuosity in the ultramicroscopy data

The tortuosity descriptor is defined as the ratio of the number of short bars ($\leq 10\%$ of maximal radius used in the radial filtration) in dimension 0 barcodes to the number of vessel segments. We divided by the number of vessel segments to ensure the contributions are topological and are not masked by an increase or decrease of vasculature. However, in the case of bevacizumab in the ultramicroscopy data, the significant decrease in number of vessel segments (*8*), which is also visually apparent when looking at examples of extracted vascular networks (see Fig. 2), leads to a seemingly contradictory increase of tortuosity (see Figure S2a). This is supported by a correlation between our tortuosity measure and the size of voids which we observed in this dataset (see Fig. S28). When considering the raw number of short bars without dividing by the number of vessel segments, we observe the expected effect of bevacizumab on tortuosity (see

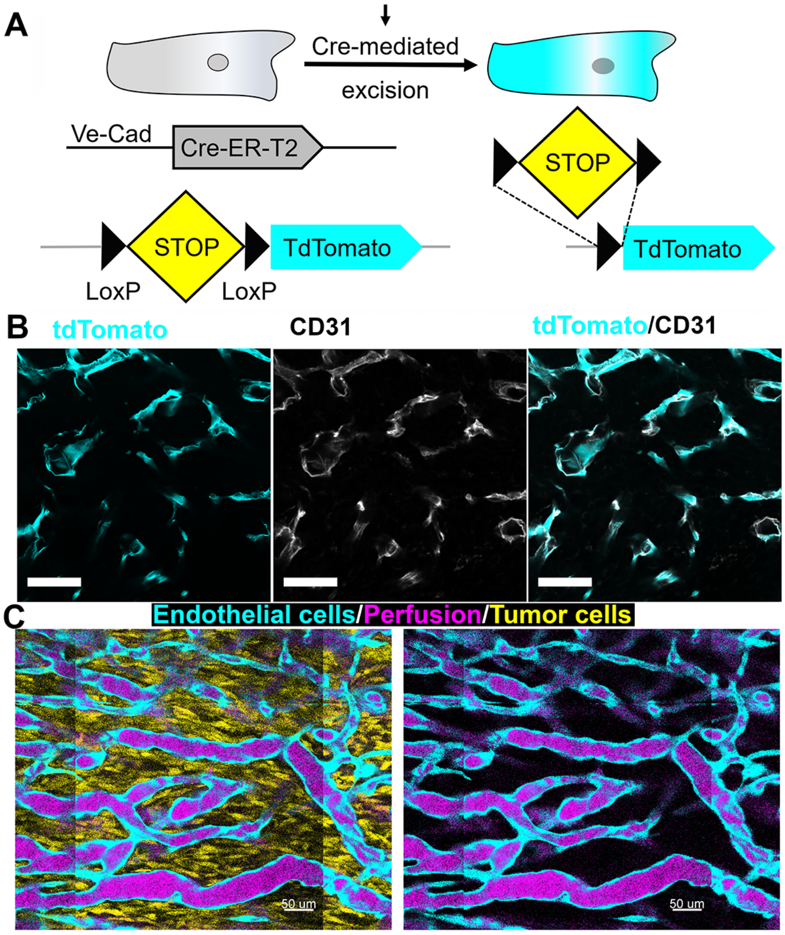

Figure S1: tdTomato expression in ECs and TECs in VE-TOM mice. A) Schematic of the Ve-Cad (Cre-ERT2) system. Administration of tamoxifen by gavaging in adult VE-TOM mice activates the Cre-LoxP system in endothelial cells inducing tdTomato expression. **B)** TECs expressing tdTomato (cyan), co-stained for CD31 (white). **C)** TECs expressing tdTomato (cyan), co-stained for CD31 (white). Representative image of a MC38 tumor. GFP positive tumor cells (yellow), TECs (cyan), and infused Qdots (purple) indicating perfused vessels. Scale bar in B and C is 50μm.

Figure S2b).

As discussed in Section Computational differences between datasets, the vascular networks in the ultramicroscopy data are much larger than in the intravital dataset. However, they are less well resolved in the xy-plane (see Data description in Materials and Methods). This has two consequences on our analysis of tortuosity: 1) the tortuosity of vessels is likely to be captured

to a lesser degree than in the intravital data, 2) the number of filtration steps needed to be able to capture tortuosity adequately would need to be significantly higher than the 500 used in the radial filtration. Indeed, example images from this data (see Fig. 2) do not appear to show strikingly tortuous vessels. Moreover, our computation of the radial filtration in 500 steps was already at the edge of computational feasibility (see Section Computational differences between datasets). Thus further refinement of the filtration is not possible. Alternatively, we can observe the coarse trends change over time without a normalisation by the number of vessel segments as shown in Figure S2b. While our topological descriptor therefore quantified a genuine and significant change in the vascular networks on the ultramicrocopy data, its interpretation here needs to be made with care.

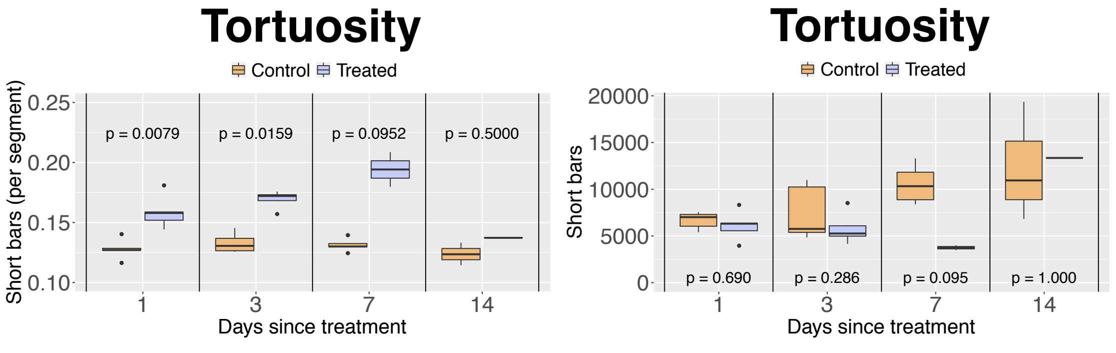

(a) (b)

Figure S2: Tortuosity in the ultramicroscopy dataset. a) Tortuosity computed as number of short bars ($\leq$ 10% of maximal radius used in the radial filtration) in dimension 0 barcode per vessel segment. **b)** Tortuosity computed as number of short bars ($\leq$ 10% of maximal radius used in the radial filtration) in dimension 0 barcode.

Alternative results figures and statistical analysis

We show alternative representations of our results from the main text. In Fig. S3 we present the results for the intravital data as mean time series for each treatment group with error bars (standard error of the mean) to highlight that our data is dynamic over time.

In Fig. S4 we present our results including p-values from our statistical analysis. We compute the (non-exact and unadjusted) p-values for the intravital data using the R function `pairwise.wilcox.test` in RSTUDIO (*76*) to perform a pairwise Wilcoxon's rank sum test between the control group and each of the treatment groups. For the ultramicroscopy data we use the function `stat_compare_means()` from the library `ggpubr` to perform Wilcoxon's rank sum test. All our tests are by default two-sided.

In Fig. S5 we present time-series of the spatio-temporal resolution of the intravital data.

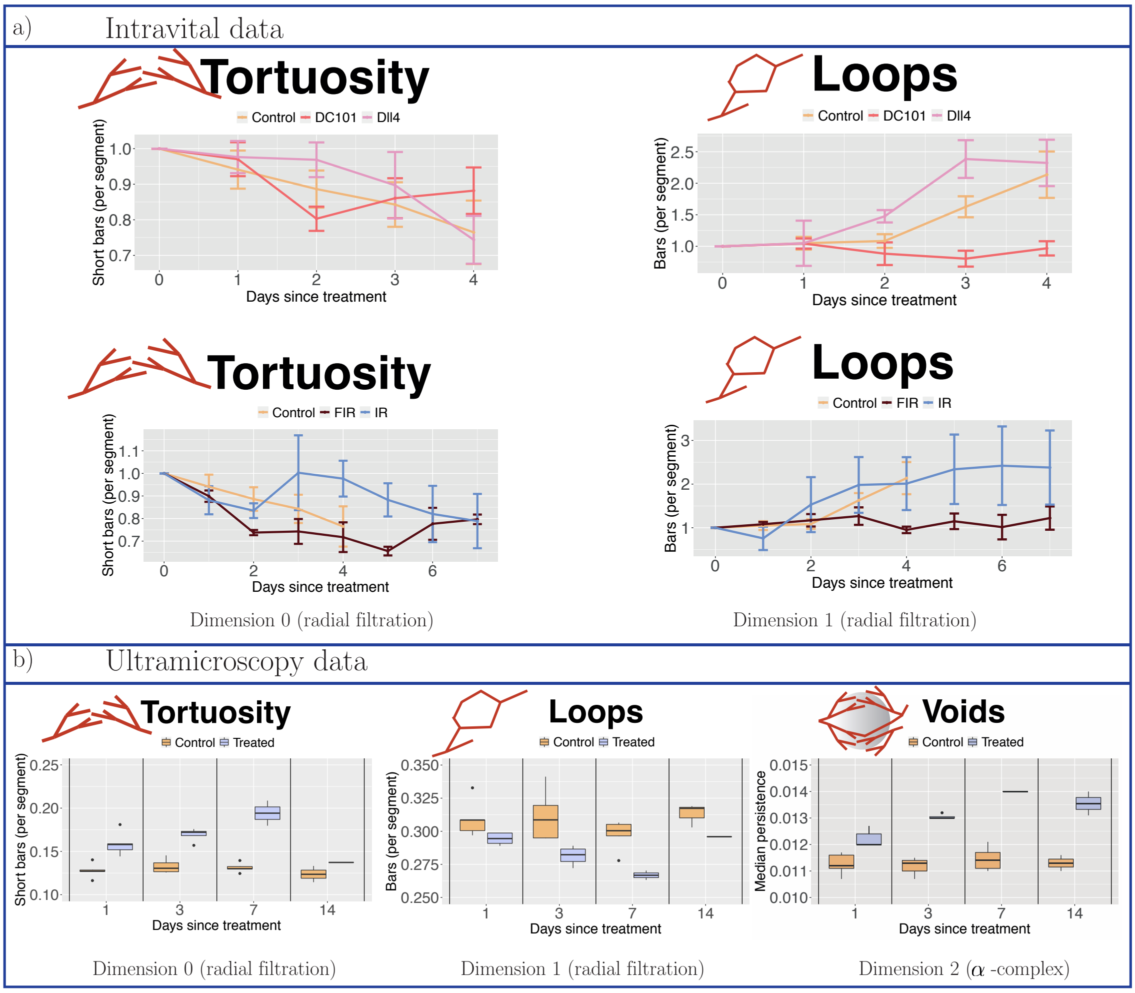

Figure S3: Topological descriptors extracted from tumour blood vessel networks treated with vascular targeting agents with known effects II. a) Intravital data results. We normalised all descriptors with respect to values on the day on which treatment is administered (day 0) or, for controls, the day on which observations commence (day 0). Data was collected from control mice (beige), mice treated with the vascular targeting agent DC101 (*37*) (dark pink), mice treated with vascular targeting agent anti-Dll4 (*39*) (light pink), mice treated with fractionated irradiation (FIR, brown), and mice treated with single dose irradiation (IR,blue). Tortuosity was computed as the ratio of short bars ($\leq 10\%$ of maximal radius used in the radial filtration) in the dimension 0 barcodes of the radial filtration to the number of vessel segments. Loops are the number of bars in the dimension 1 barcodes of the radial filtration per vessel segment in the network. **b)** Ultramicroscopy data results. Due to the snapshot nature of the data (one time point per tumour), all reported topological descriptors are raw values. Data was collected from control mice (beige) and mice treated with bevacizumab (purple). We computed tortuosity values and the number of vessel loops per vessel segment, in the same way as for the intravital data. We also determined the size of voids (avascular regions) by computing the median length of bars in the dimension 2 barcodes of the α-complex filtration.

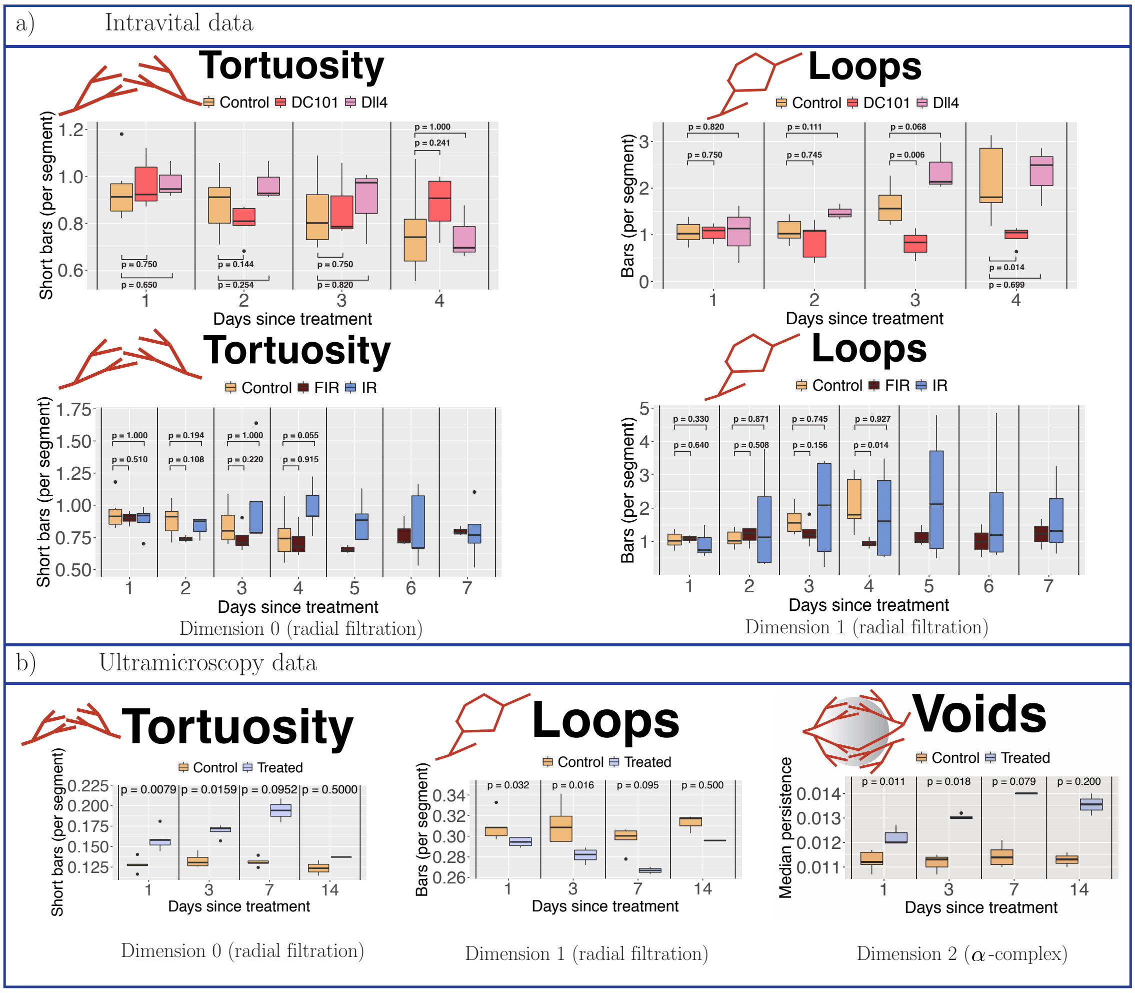

Figure S4: Topological descriptors extracted from tumour blood vessel networks treated with vascular targeting agents with known effects III. a) Intravital data results. We normalised all descriptors with respect to values on the day on which treatment is administered (day 0) or, for controls, the day on which observations commence (day 0). Data was collected from control mice (beige), mice treated with the vascular targeting agent DC101 (*37*) (dark pink), mice treated with vascular targeting agent anti-Dll4 (*39*) (light pink), mice treated with fractionated irradiation (FIR, brown), and mice treated with single dose irradiation (IR,blue). Tortuosity was computed as the ratio of short bars ($\leq 10\%$ of maximal radius used in the radial filtration) in the dimension 0 barcodes of the radial filtration to the number of vessel segments. Loops are the number of bars in the dimension 1 barcodes of the radial filtration per vessel segment in the network. **b)** Ultramicroscopy data results. Due to the snapshot nature of the data (one time point per tumour), all reported topological descriptors are raw values. Data was collected from control mice (beige) and mice treated with bevacizumab (purple). We computed tortuosity values and the number of vessel loops per vessel segment, in the same way as for the intravital data. We also determined the size of voids (avascular regions) by computing the median length of bars in the dimension 2 barcodes of the α-complex filtration.

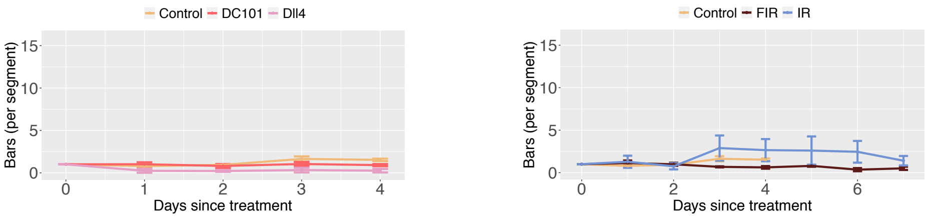

(a) Radial interval I.

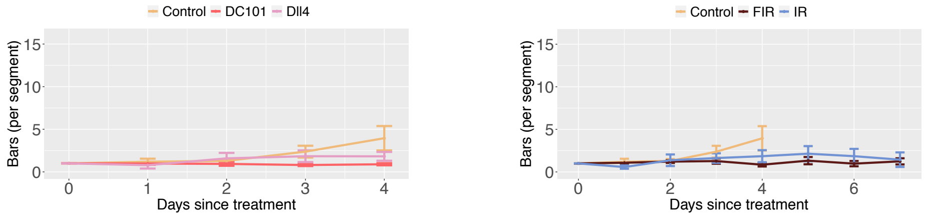

(b) Radial interval II.

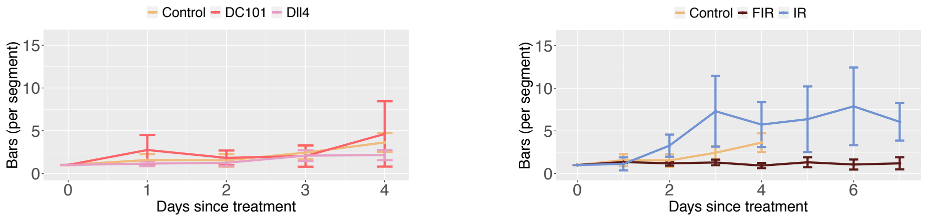

(c) Radial interval III.

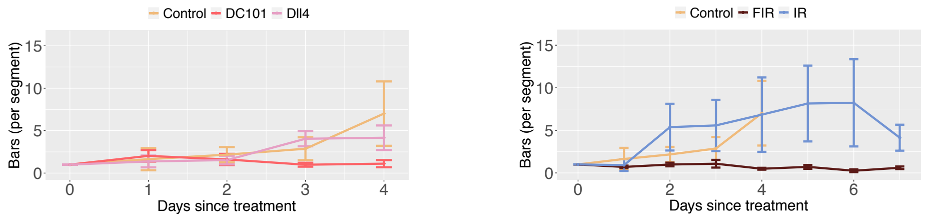

(d) Radial interval IV.

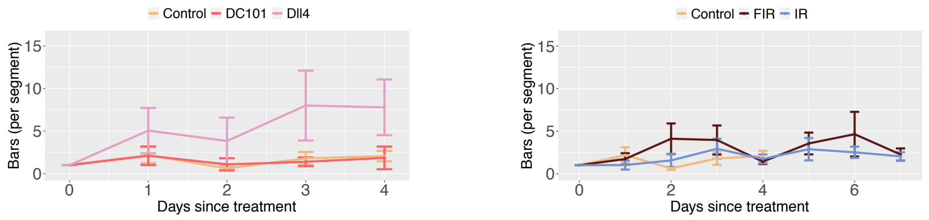

(e) Radial interval V.

Figure S5: Mean time series of the normalised number of loops per vessel segment for different filtration intervals in the intravital dataset. We show the mean of the number of loops normalised by day 0 and standard error of the mean in different radial intervals. Interval I corresponds to the radial region closest to the tumour centre, while Interval V represents parts of the vessel network that are farthest away from the tumour centre. We separate the treatments into two groups to facilitate the distinction of the trends.

Additional results and statistical analysis

Intravital data: DC101 versus anti-Dll4. We present statistical analysis on the control group and treatment groups DC101 and anti-Dll4 in the intravital data. We use the function `stat_compare_means()` from the library `ggpubr` to compute Kruskal-Wallis test p-values for tortuosity (see Fig. S6), number of loops per vessel segment (see Fig. S7) as well as the following standard measures for vascular networks: number of vessel segments (see Fig. S8), number of branching points (see Fig. S9), average vessel diameter (see Fig. S10), maximal diameter (see Fig. S11), average (mean) vessel length (see Fig. S12), maximal vessel length (see Fig. S13), average (mean) chord length ratio (see Fig. S14), average (mean) sum of angles metric (see Fig. S15), and length-diameter ratio (see Fig. S16). All values are normalised by day 0 of observation/treatment and were obtained from the PYTHON code package UNET-CORE (*44*).

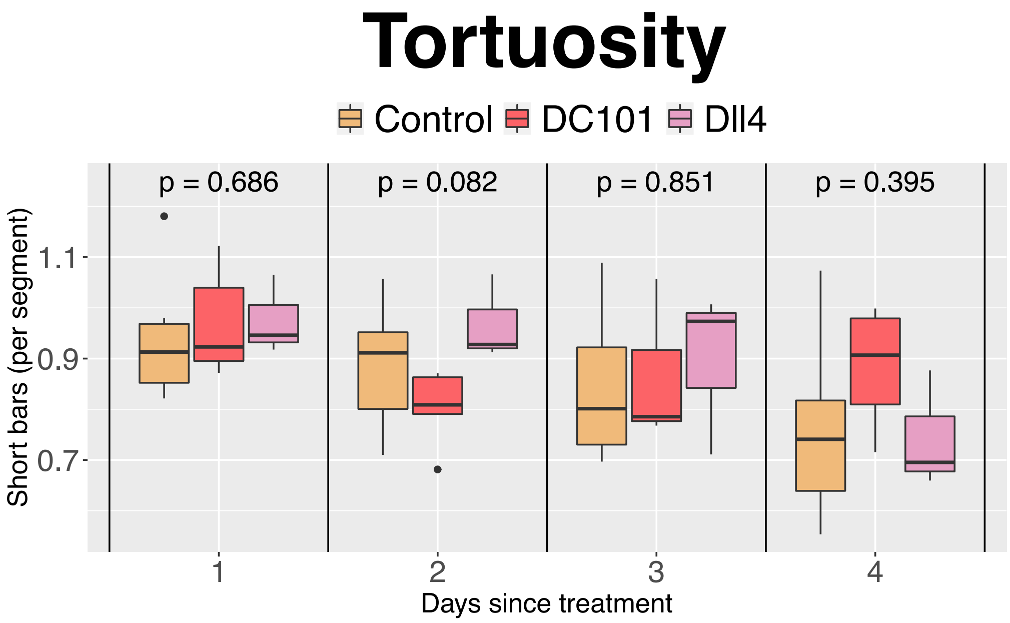

Figure S6: Box plot showing the number of short bars in the dimension 0 barcode of the radial filtration divided by the number of vessel segments. The values are normalised by day 0 of initial treatment for all treatment regimes to facilitate comparisons of trends over time. We show group level p-values according to the Kruskal-Wallis test.

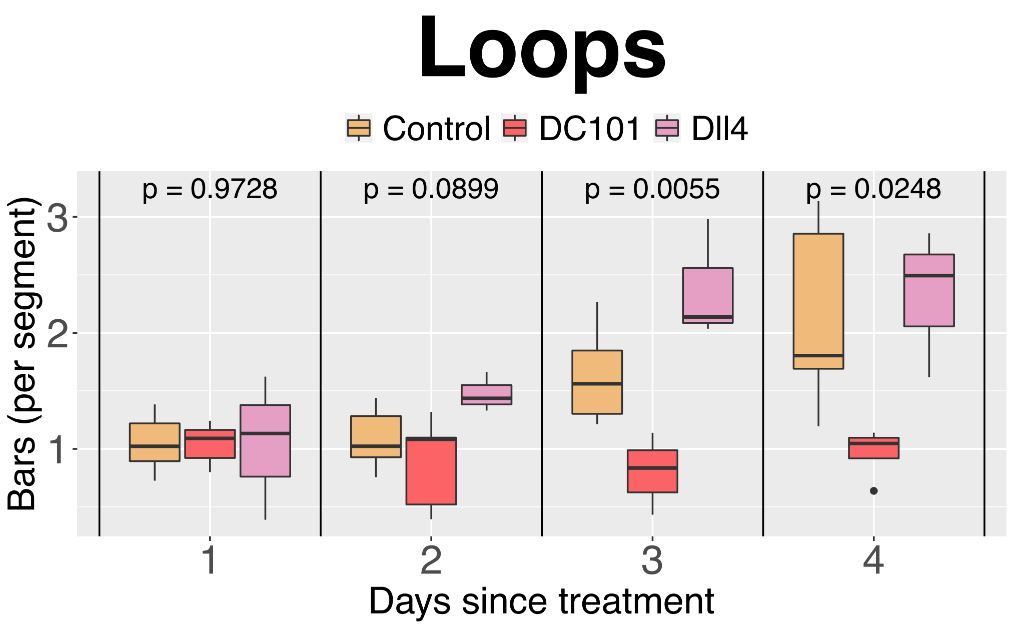

Figure S7: Box plot showing the number of bars in the dimension 1 barcode of the radial filtration divided by the number of vessel segments. The values are normalised by day 0 of initial treatment for all treatment regimes to facilitate comparisons of trends over time. We show group level p-values according to the Kruskal-Wallis test.

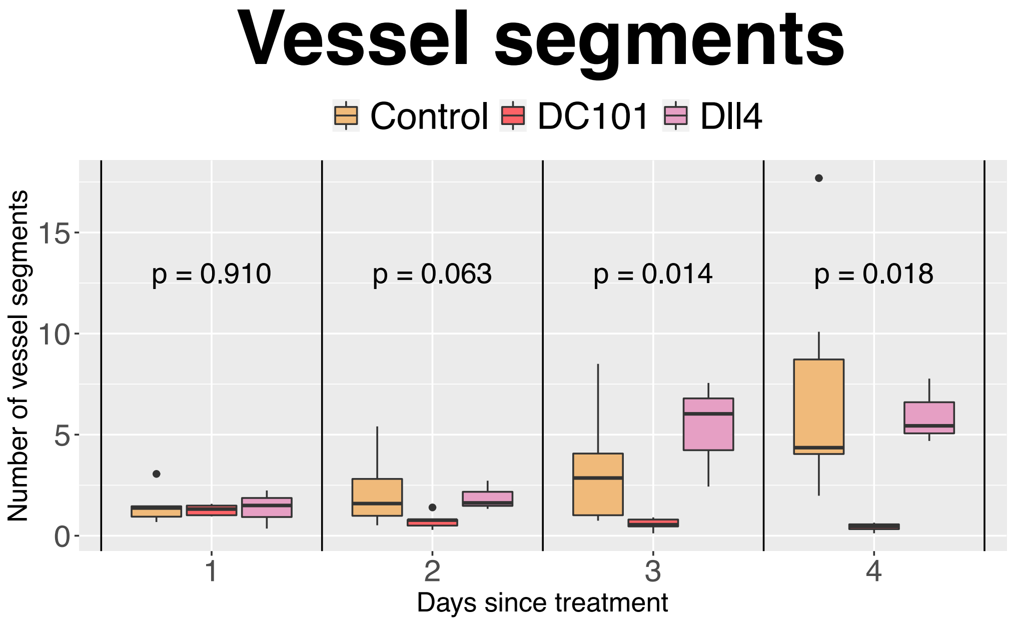

Figure S8: Box plot showing the number of vessel segments. The values are normalised by day 0 of initial treatment for all treatment regimes to facilitate comparisons of trends over time. We show group level p-values according to the Kruskal-Wallis test.

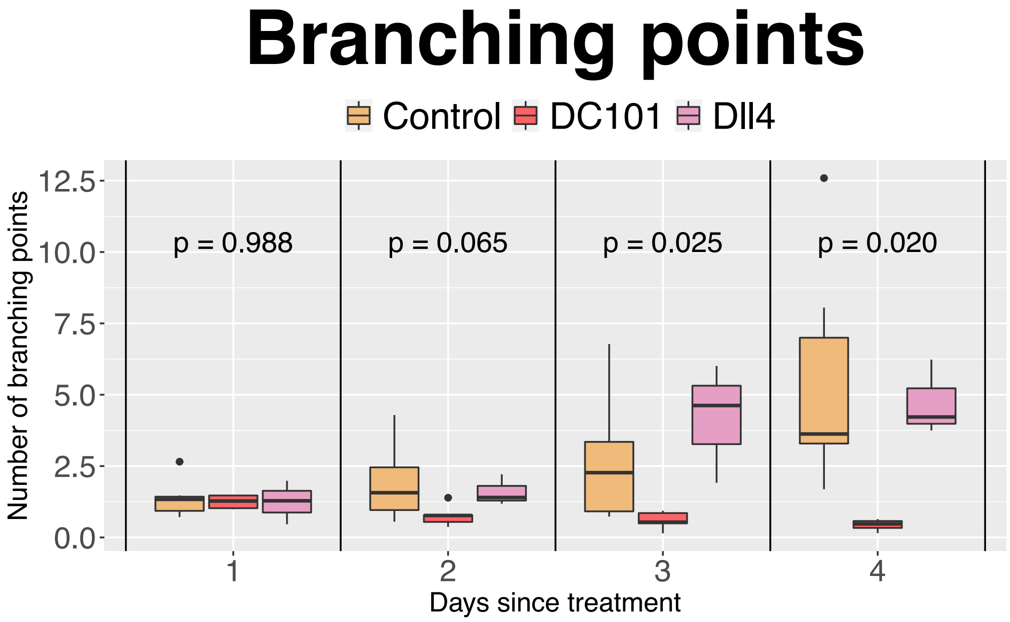

Figure S9: Box plot showing the number of branching points. The values are normalised by day 0 of initial treatment for all treatment regimes to facilitate comparisons of trends over time. We show group level p-values according to the Kruskal-Wallis test.

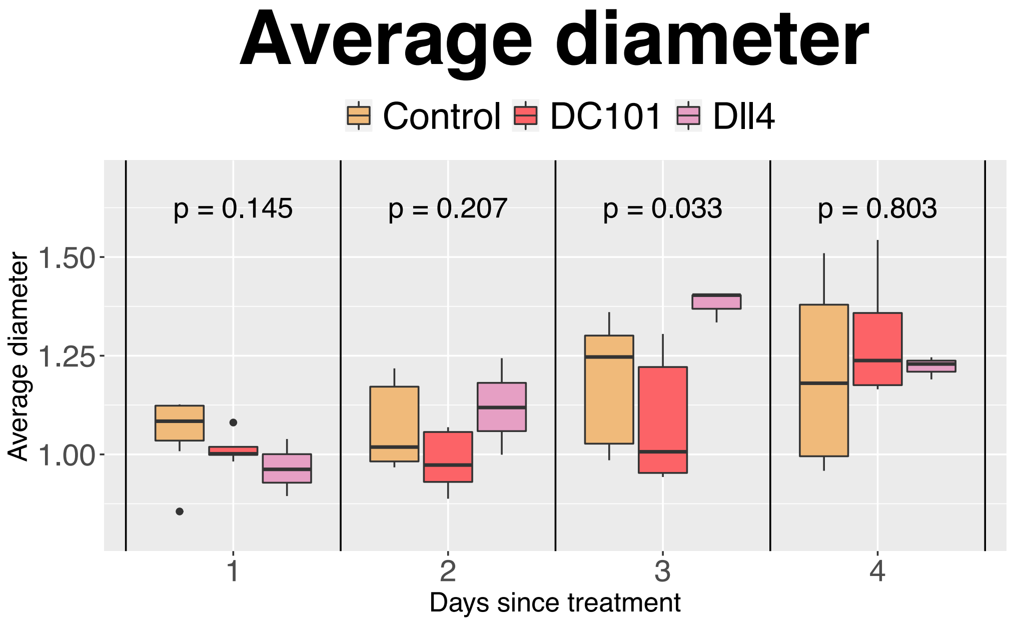

Figure S10: Box plot showing the average (mean) vessel diameter. The values are normalised by day 0 of initial treatment for all treatment regimes to facilitate comparisons of trends over time. We show group level p-values according to the Kruskal-Wallis test.

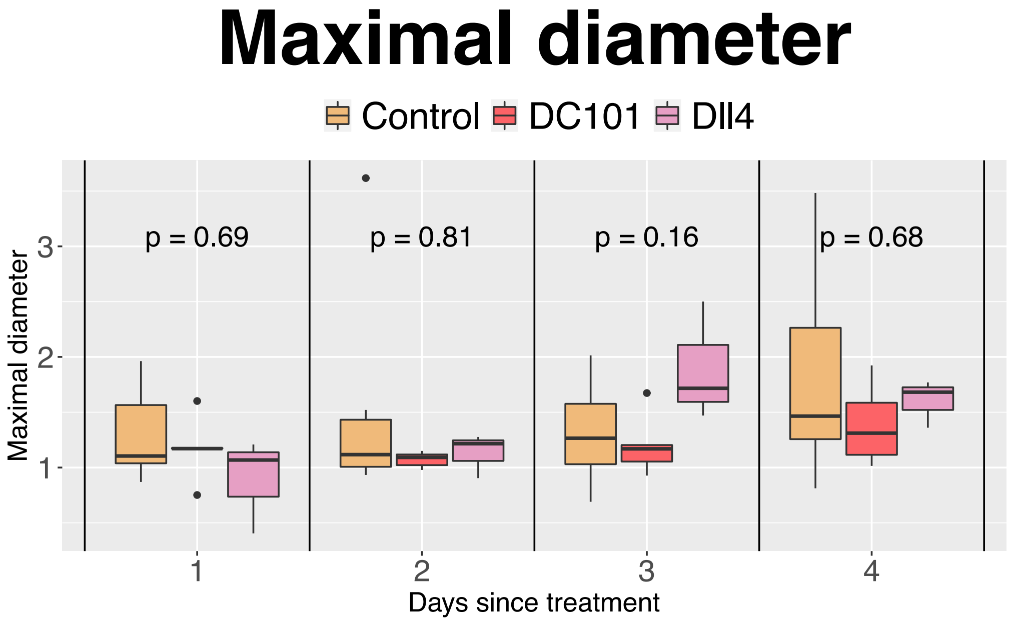

Figure S11: Box plot showing the maximal vessel diameter. The values are normalised by day 0 of initial treatment for all treatment regimes to facilitate comparisons of trends over time. We show group level p-values according to the Kruskal-Wallis test.

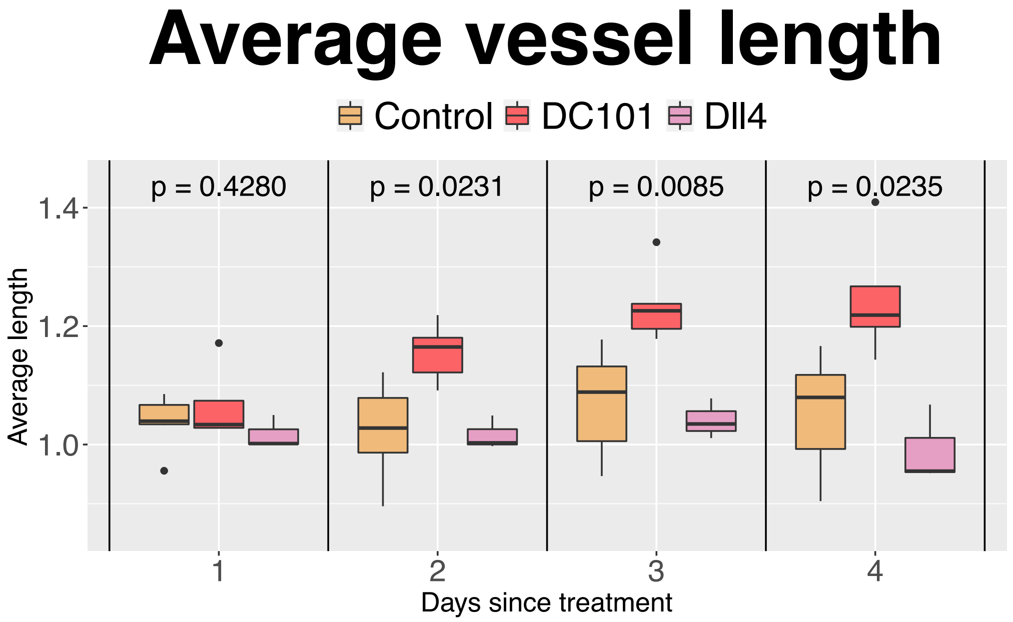

Figure S12: Box plot showing the average (mean) vessel length. The values are normalised by day 0 of initial treatment for all treatment regimes to facilitate comparisons of trends over time. We show group level p-values according to the Kruskal-Wallis test.

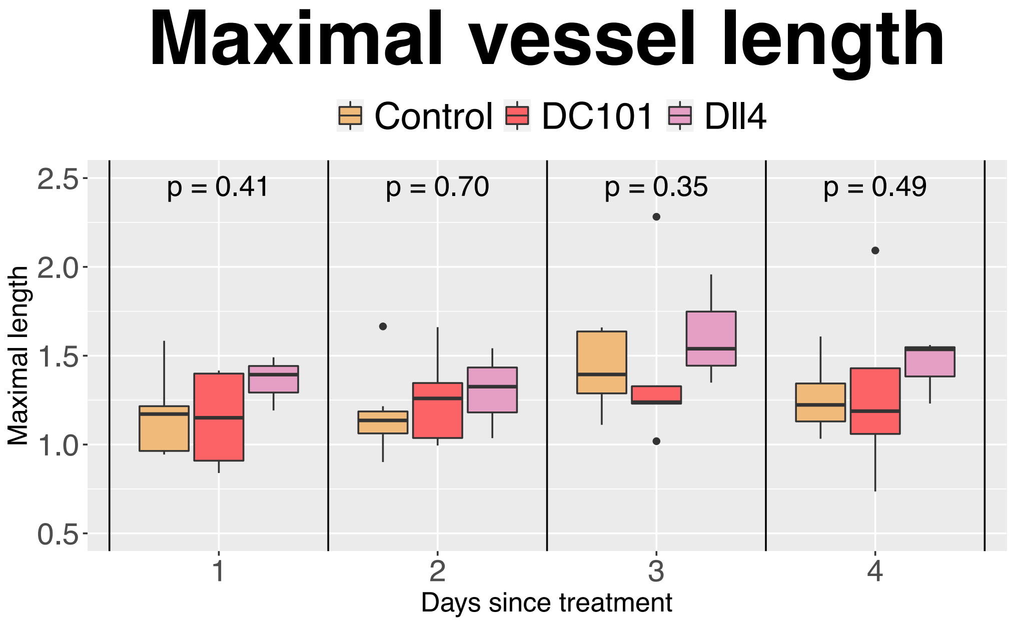

Figure S13: Box plot showing the average (mean) vessel length. The values are normalised by day 0 of initial treatment for all treatment regimes to facilitate comparisons of trends over time. We show group level p-values according to the Kruskal-Wallis test.

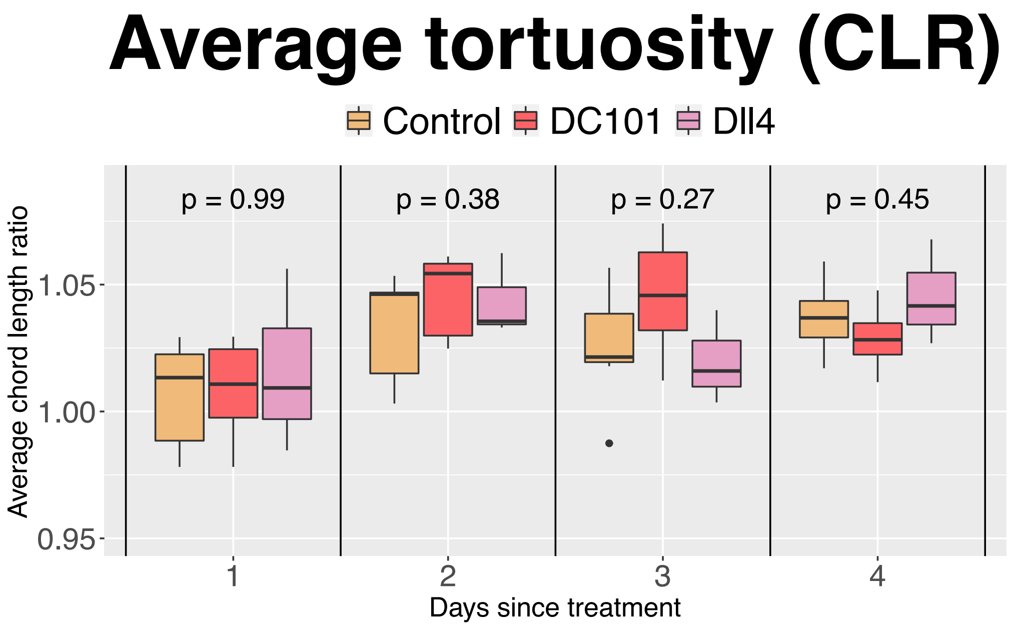

Figure S14: Box plot showing the average (mean) chord length ratio. The values are normalised by day 0 of initial treatment for all treatment regimes to facilitate comparisons of trends over time. We show group level *p*-values according to the Kruskal-Wallis test.

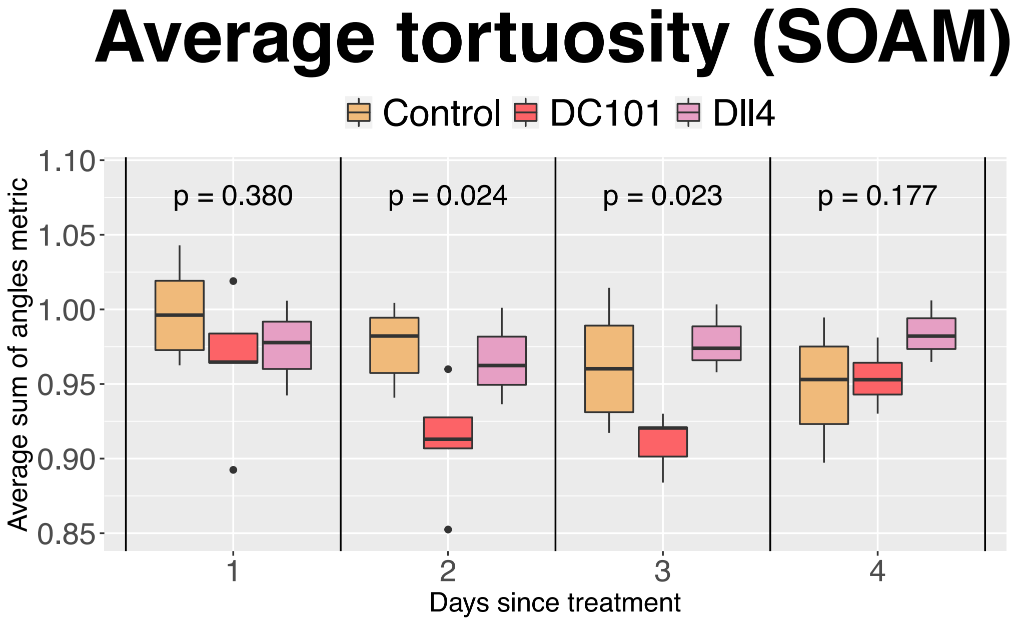

Figure S15: Box plot showing the average (mean) sum of angles metric. The values are normalised by day 0 of initial treatment for all treatment regimes to facilitate comparisons of trends over time. We show group level p-values according to the Kruskal-Wallis test.

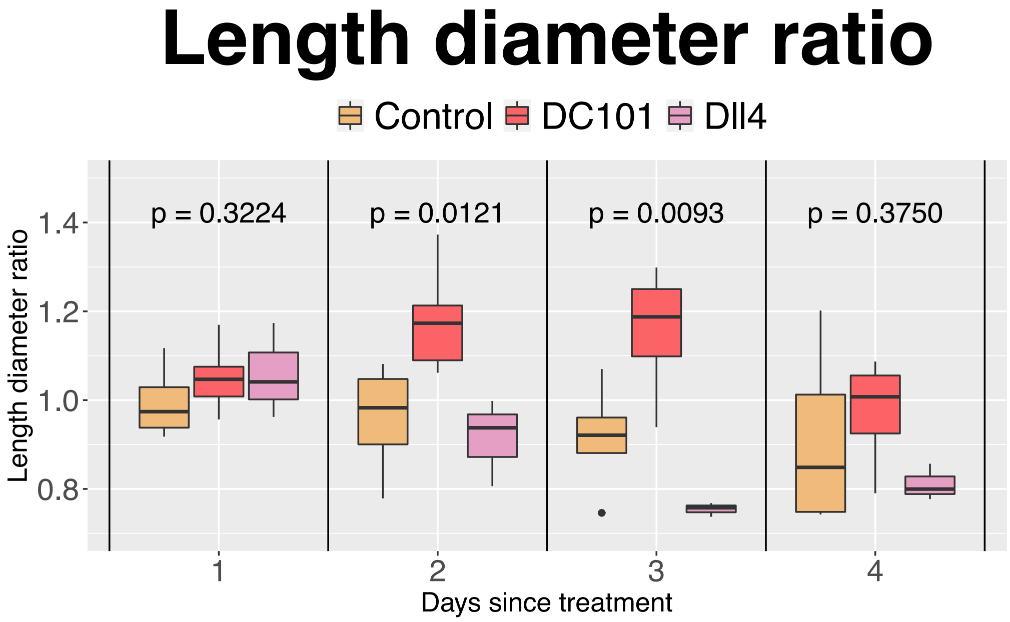

Figure S16: Box plot showing the length to diameter ratio. The values are normalised by day 0 of initial treatment for all treatment regimes to facilitate comparisons of trends over time. We show group level p-values according to the Kruskal-Wallis test.

Intravital data: single dose irradiation versus fractionated dose irradiation. We present statistical analysis on the control group and radiation treatment groups IR (single-dose irradiation) and FIR (fractionated-dose irradiation) in the intravital data. We use the function `stat_compare_means()` from the library `ggpubr` to compute Kruskal-Wallis test p-values for tortuosity (see Fig. S17) and number of loops per vessel segment (see Fig. S18). All values are normalised by day 0 of observation/treatment.

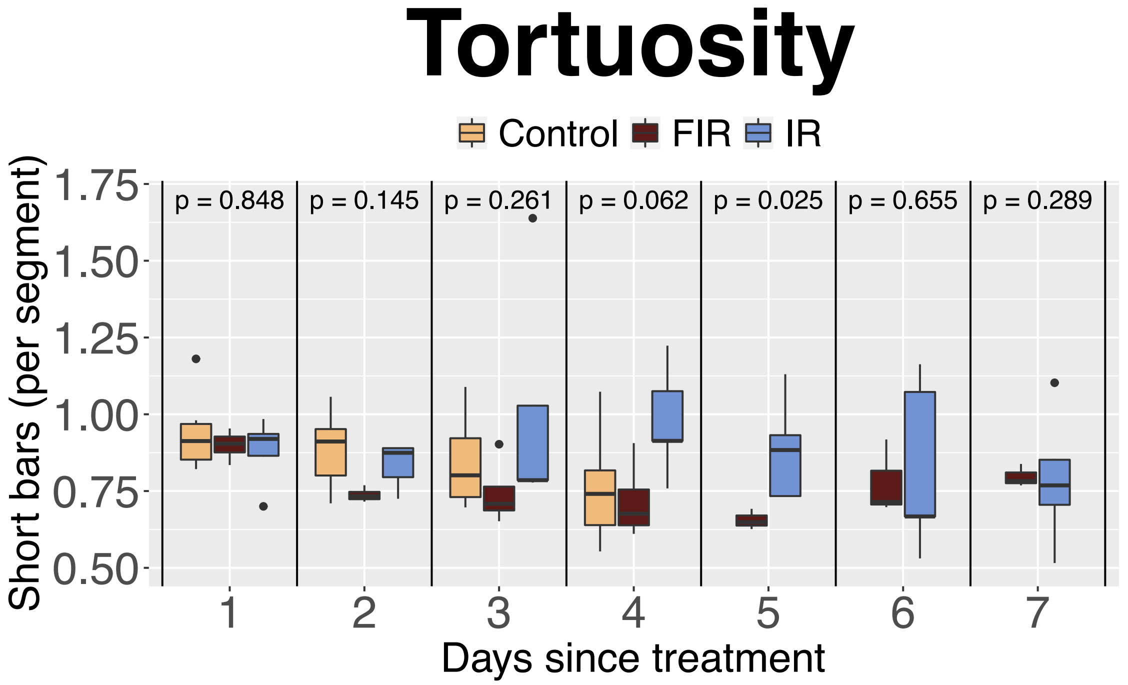

Figure S17: Box plot showing the number of short bars in the dimension 0 barcode of the radial filtration divided by the number of vessel segments. The values are normalised by day 0 of initial treatment for all treatment regimes to facilitate comparisons of trends over time. We show group level p-values according to the Kruskal-Wallis test.

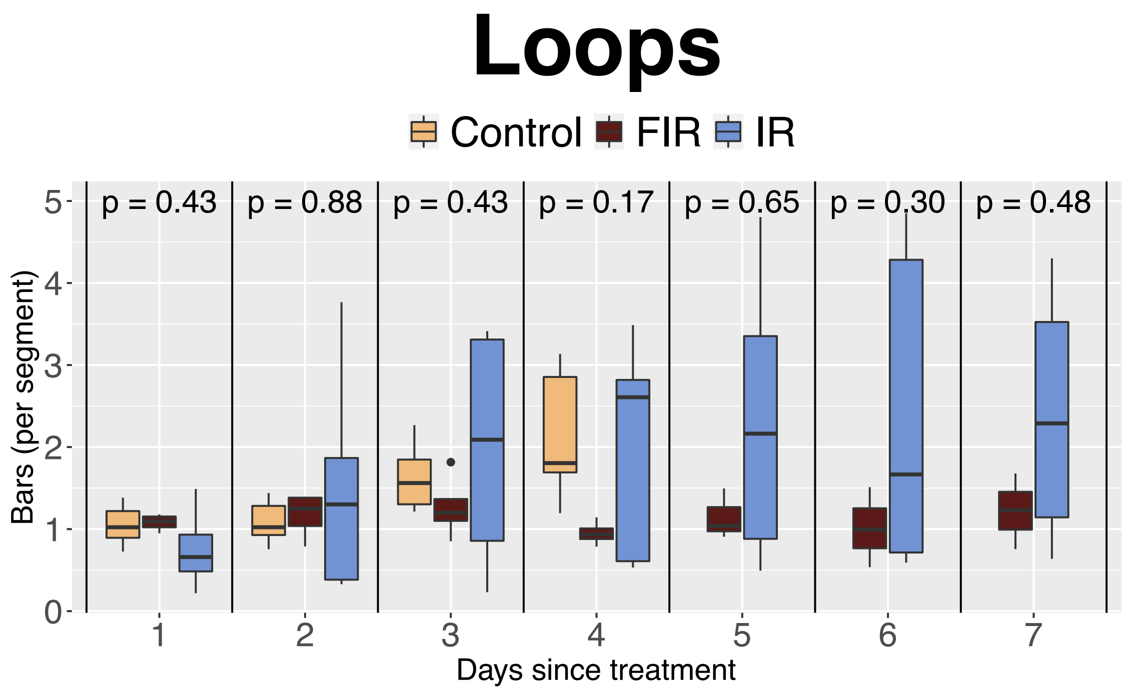

Figure S18: Box plot showing the number of bars in the dimension 1 barcode of the radial filtration divided by the number of vessel segments. The values are normalised by day 0 of initial treatment for all treatment regimes to facilitate comparisons of trends over time. We show group level p-values according to the Kruskal-Wallis test.

Intravital data: all treatment groups. We present statistical analysis to determine whether at least one of the treatment groups in the intravital data behaves significantly differently to the others in Fig. S19 for our extracted tortuosity measure and in Fig. S20 for the number of loops per vessel segment. All values are normalised by day 0 of observation/treatment. We compute the (non-exact) p-values for the using the R function `kruskal.test()` to compute Kruskal-Wallis in RSTUDIO (*76*). We further present the same analysis for parameters not shown in the main text, i.e. for voids in Fig. S21 and maximal radii used in the radial filtration (i.e. an approximation of the tumour radii) in Fig. S22. Again, all values are normalised by day 0 of observation/treatment. We note that both of these parameters do not show significant differences between treatment groups. In the case of the voids in the intravital dataset this can be explained by the the low penetration depth of the imaging.

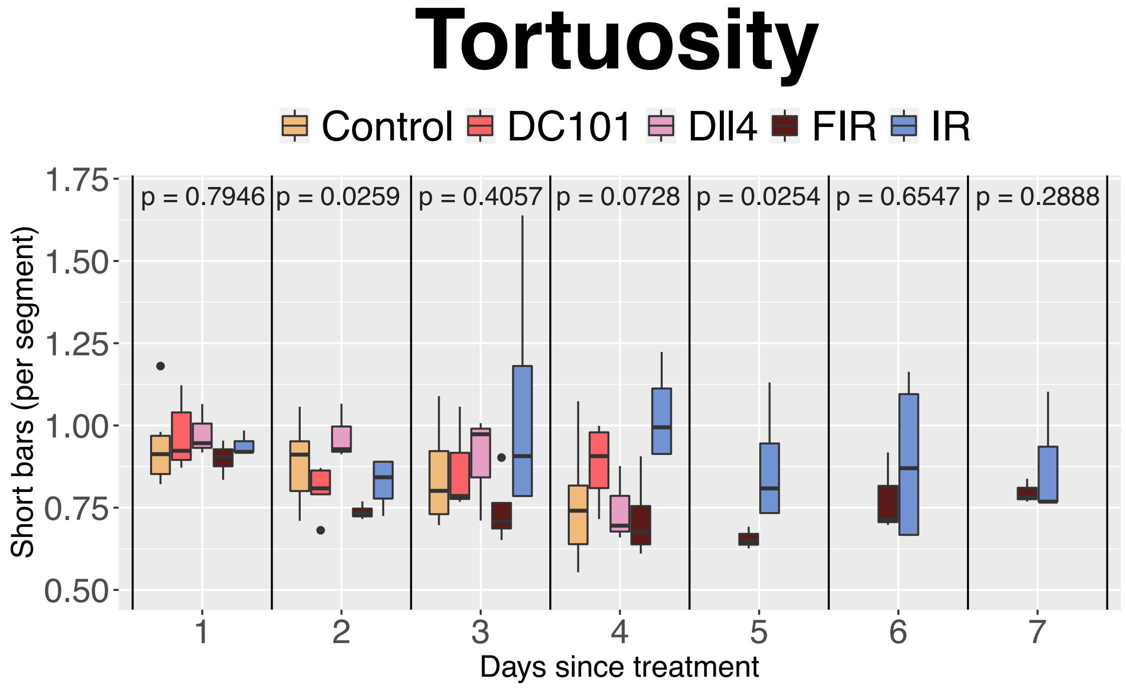

Figure S19: Box plot showing the number of short bars in the dimension 0 barcode of the radial filtration divided by the number of vessel segments. The values are normalised by day 0 of initial treatment for all treatment regimes to facilitate comparisons of trends over time. We show group level p-values according to the Kruskal-Wallis test.

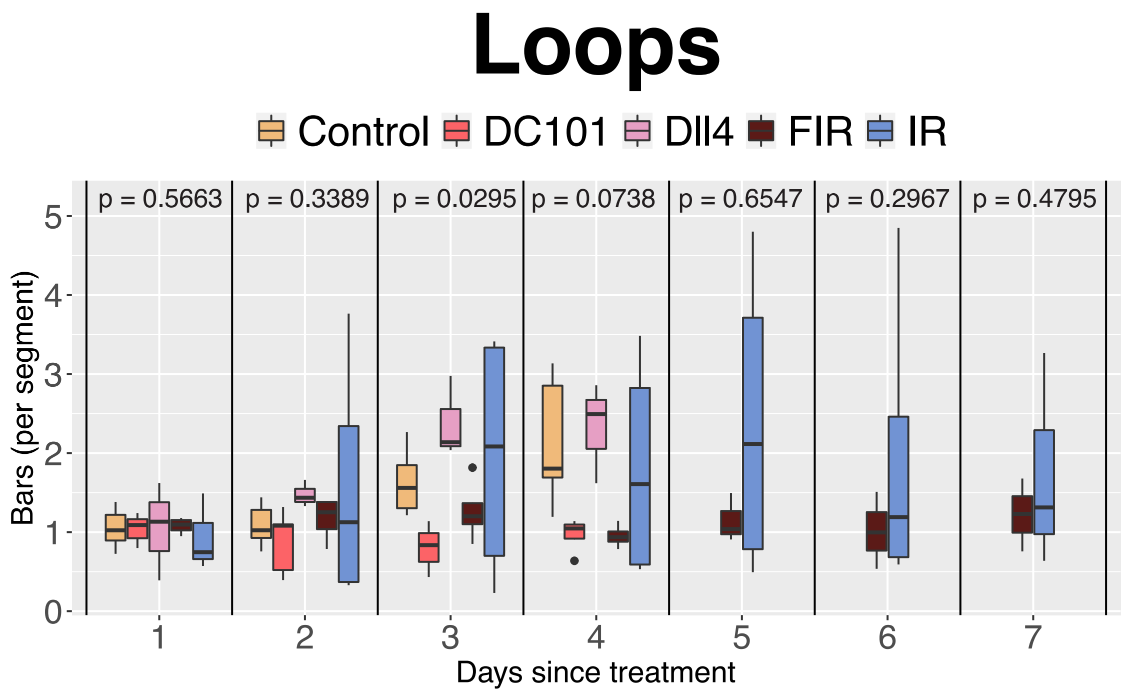

Figure S20: Box plot showing the number of bars in the dimension 1 barcode of the radial filtration divided by the number of vessel segments. The values are normalised by day 0 of initial treatment for all treatment regimes to facilitate comparisons of trends over time. We show group level p-values according to the Kruskal-Wallis test.

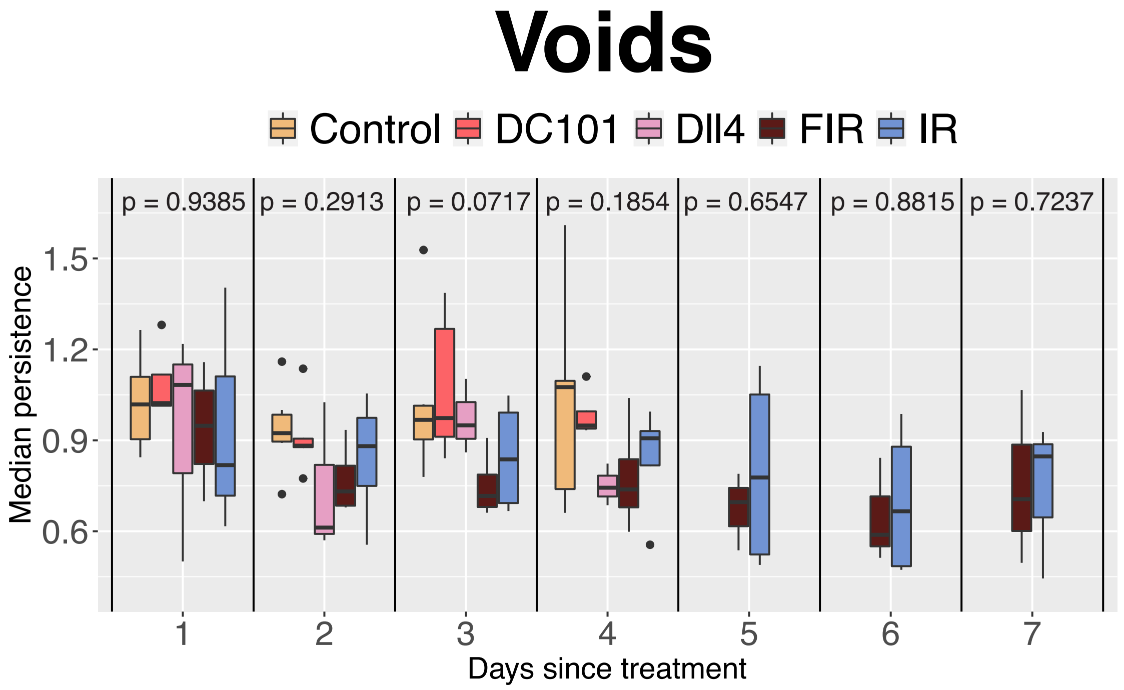

Figure S21: Box plot showing median persistence of bars in the dimension 2 barcode of the α-complex filtration. The values are normalised by day 0 of initial treatment for all treatment regimes to facilitate comparisons of trends over time. We show p-values according to the Kruskal-Wallis test.

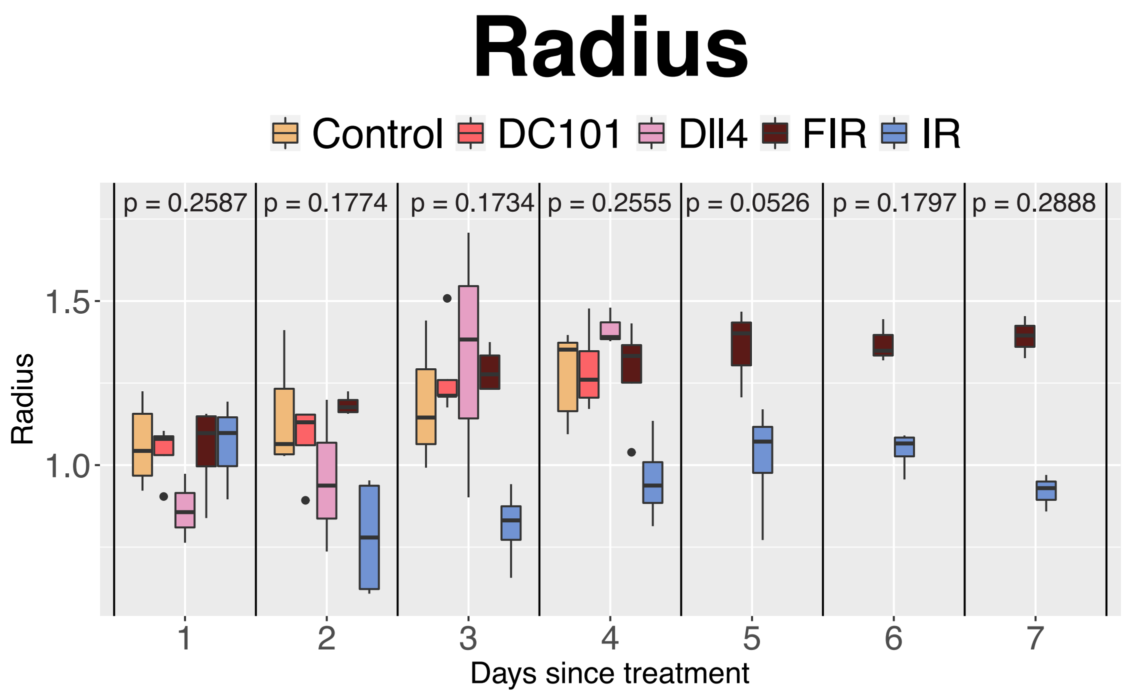

Figure S22: Box plot showing the maximal radius attained in the radial filtration. The values are normalised by day 0 of initial treatment for all treatment regimes to facilitate comparisons of trends over time. We show p-values according to the Kruskal-Wallis test.

Finally, we present a correlation analysis between parameters that are conventionally extracted from vascular networks and our topological parameters in Fig. S23. We compute pairwise Pearson correlation using the library `hmisc` and plot our results including a complete linkage clustering dendrogramme of the parameters using the library `corrplot` in RSTUDIO (*76*).

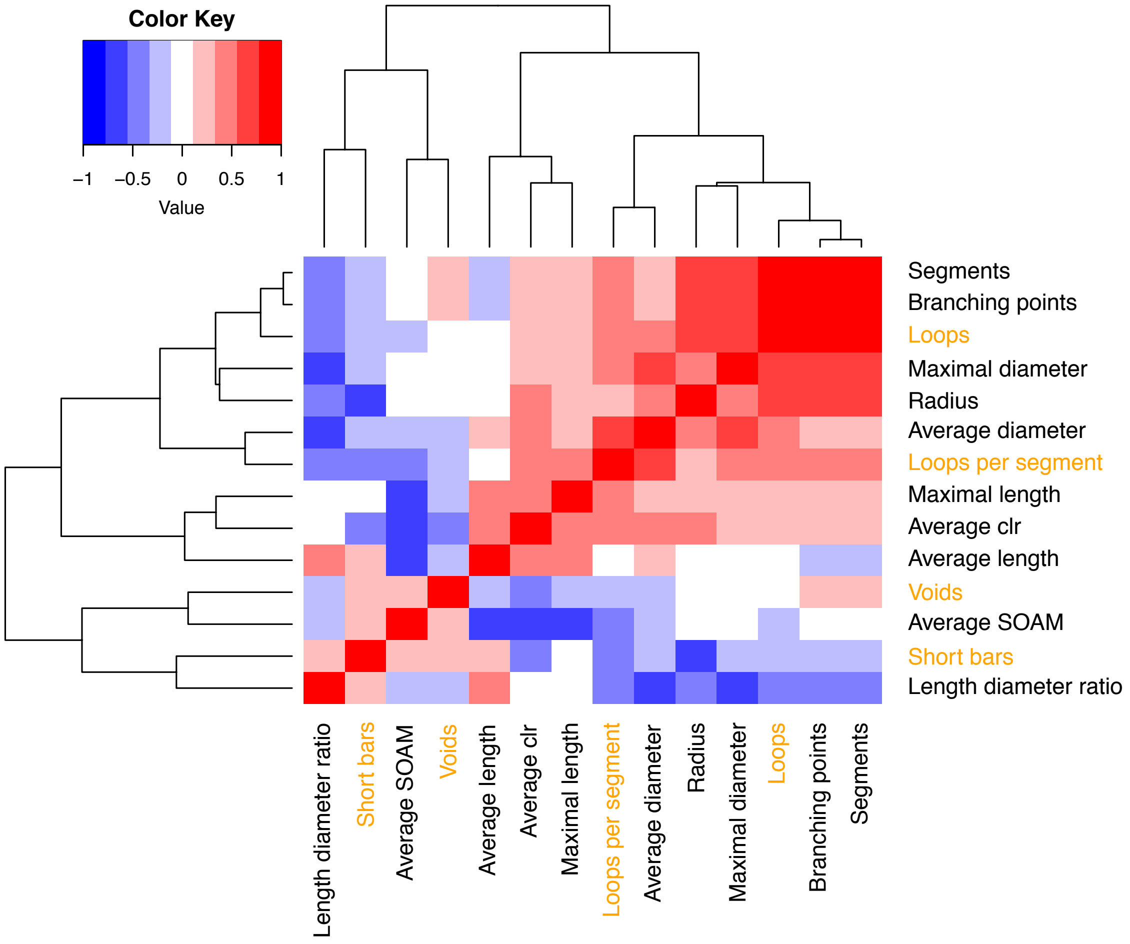

Figure S23: Heatmap displaying the pairwise Pearson correlation coefficients between different vascular characteristics derived from the intravital data. The dendrogramme represents complete linkage clustering using the Euclidean distance measure. We consider the following vascular characteristics: number of vessel segments (i.e. number of edges), number of branching points (i.e. number of nodes), number of vessel loops, maximal vessel diameter, maximal radius used in the radial filtration, average vessel diameter, number of vessel loops per vessel segment, maximal vessel length, average chord length ratio (clr), average vessel length, median persistence of bars in dimension 2 barcodes (voids), average sum of angles measure (SOAM), number of short bars per vessel segment in the dimension 0 barcodes, vessel length/diameter ratio. We highlight the topological measures in orange including both the number of loops and number of loops per vessel segment to highlight the effect of the normalisation.

Ultramicroscopy data. We present box plots of the tumour volume as determined by Dobosz *et al.* (*8*) in Fig. S24 and the maximal radii used in the radial filtration in Fig. S25. We compute the (non-exact) p-values using function `stat_compare_means` from the library `ggpubr` in RSTUDIO (*76*) to perform a pairwise Wilcoxon's rank sum test between the control group and the treatment group. All our tests are by default two-sided. We further show the spatio-temporal resolution of the number of loops in the ultramicroscopy data in Fig. S26. We do not find any marked differences in either treatment group in different spherical shells around the tumour centres.

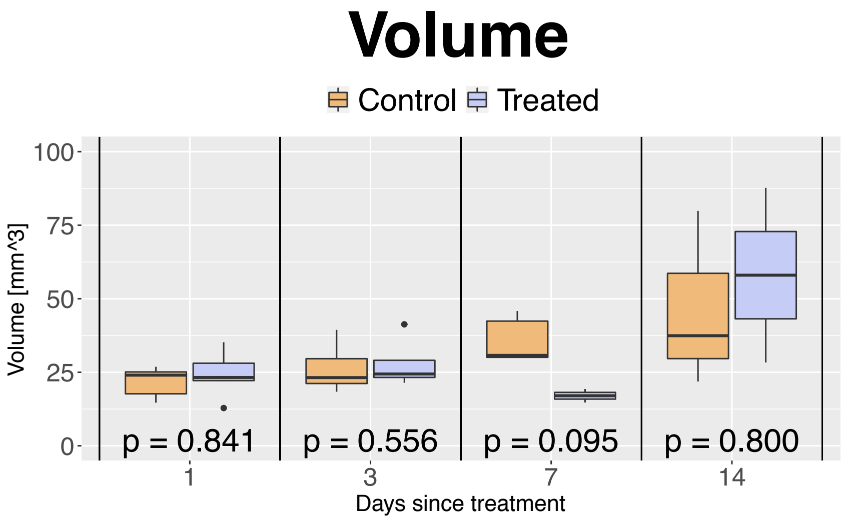

Figure S24: Box plot showing tumour volume as determined by Dobosz *et al.* (*8*). We show p-values according to Wilcoxon's rank sum test.

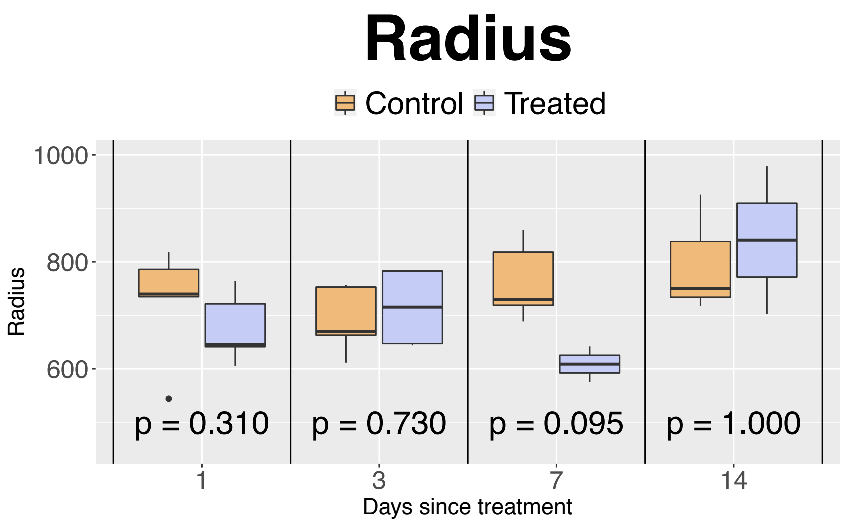

Figure S25: Box plot showing the maximal radius attained in the radial filtration. We show p-values according to Wilcoxon's rank sum test.

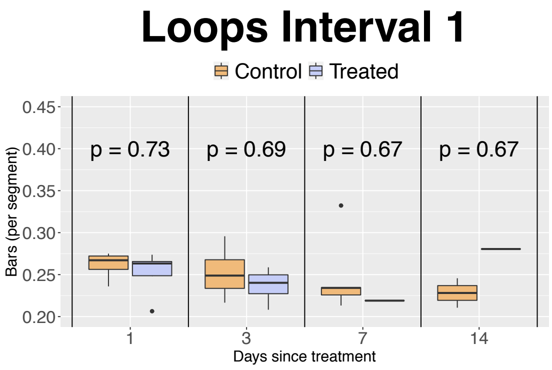

(a) Radial interval I.

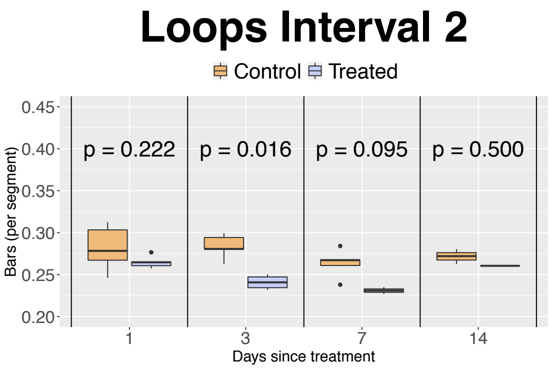

(b) Radial interval II.

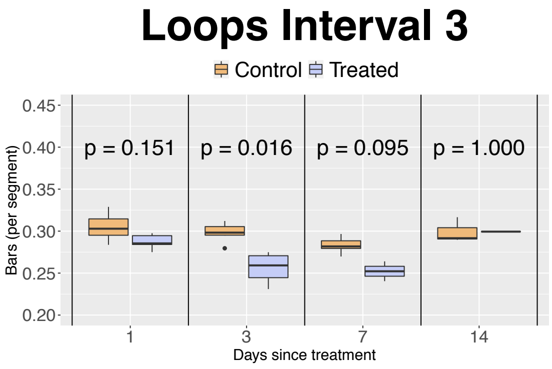

(c) Radial interval III.

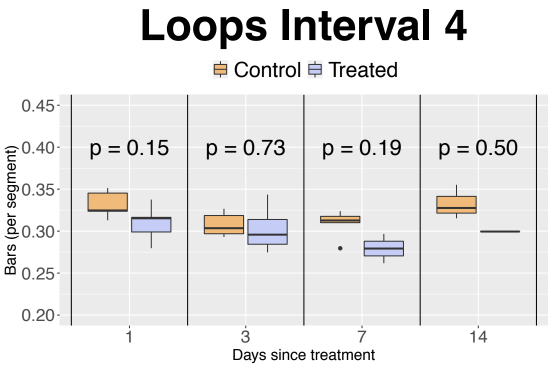

(d) Radial interval IV.

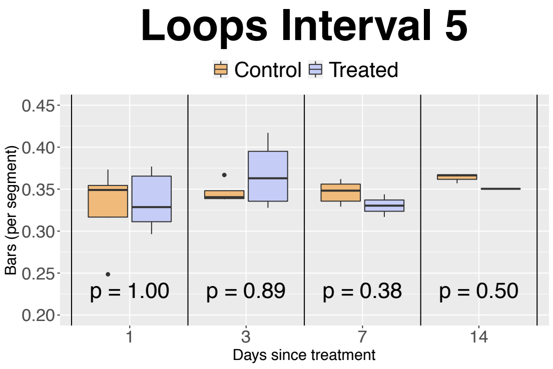

(e) Radial interval V.

Figure S26: Number of loops per vessel segment for different filtration intervals in the ultramicroscopy dataset. We show box plots of the number of loops per vessel segment. Interval I corresponds to the radial region closest to the tumour centre, while Interval V represents parts of the vessel network that are farthest away from the tumour centre. We show p-values according to Wilcoxon's rank sum test.

We present the distribution of loops in the ultramicroscopy data relative to the tumour radii in Fig. S27. We apply a Anderson-Darling test using the function `ad.test()` from the library `ksamples` in RSTUDIO (*76*) to the different time points and treatment groups to determine whether the samples within one groups come from a common (unspecified) distribution. We do not find this to be the case in any of the groups for any time point.

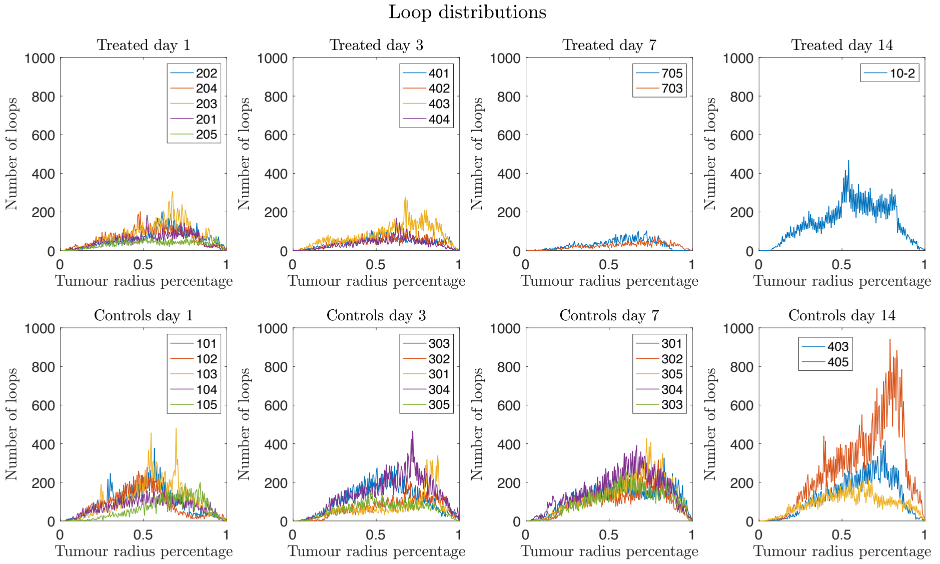

Figure S27: Spatial distribution of the number of loops in the ultramicroscopy data. We show the distribution of loops in individual tumours grouped by treatment regime (top row: bevacicumab treated tumours; bottom row: control tumours) and time points (column 1: day 1 after treatment; column 2: day 2 after treatment; column 3: day 3 after treatment; column 4: day 4 after treatment). The horizontal axis represents the radial distance to the tumour centre normalised by tumour radius.

Finally, we present a correlation analysis between parameters that were extracted by Dobosz *et al.* (*8*) and our topological parameters in Fig. S23. We also include the number of segments and branching points determined by our extraction of the vessel networks with UNET-CORE (*44*). Both of these standard parameters correlate strongly with the same parameters extracted by Dobosz *et al.* (*8*). We compute pairwise Pearson correlation using the library `hmisc` and plot our results including a complete linkage clustering dendrogramme of the parameters using the library `corrplot` in RSTUDIO (*76*).

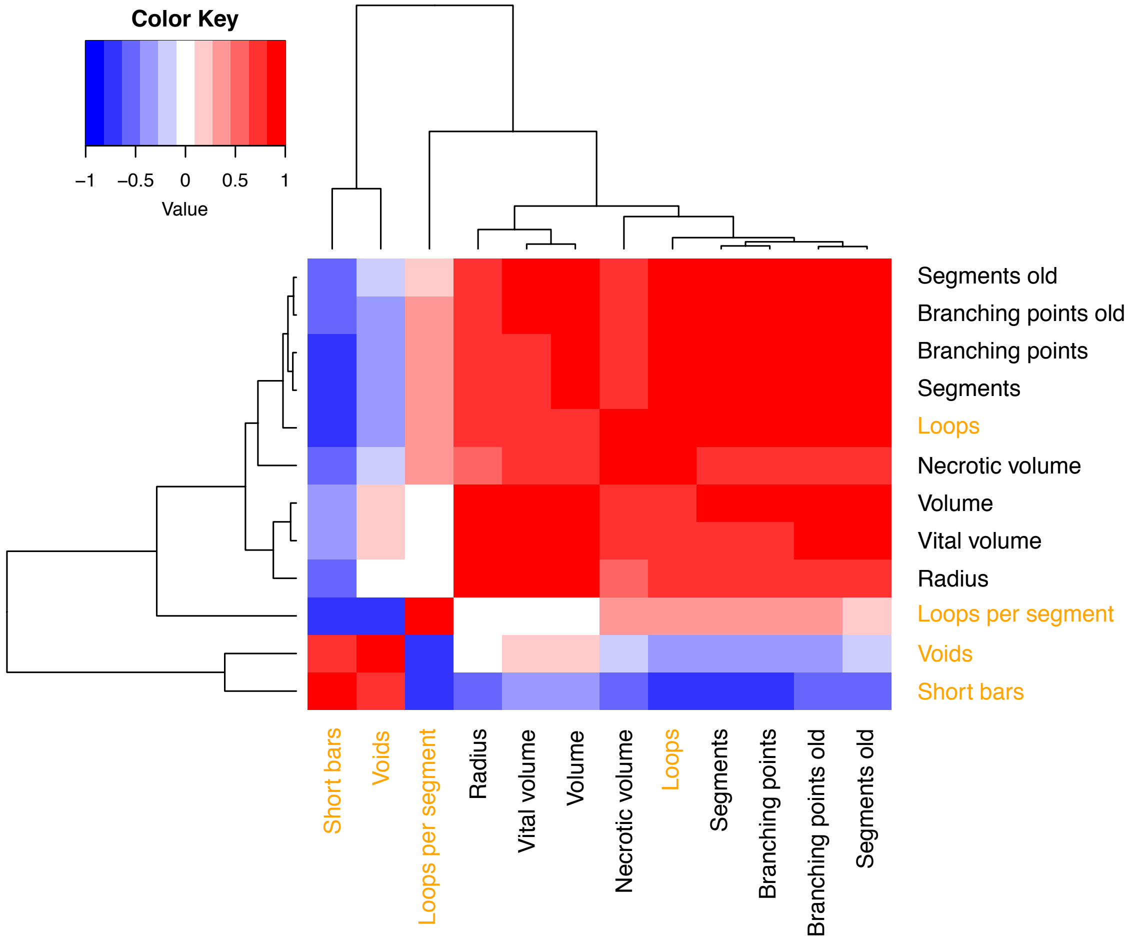

Figure S28: Heatmap displaying the pairwise Pearson correlation coefficients between different vascular characteristics derived from the ultramicroscopy data. The dendrogramme represents complete linkage clustering using the Euclidean distance measure. We consider the following vascular characteristics: number of vessel segments as computed by (*8*) (segments old), number of branching points as computed by (*8*) (branching points old), number of branching points as computed by *unet*, number of vessel segments as computed by *unet*, number of vessel loops, necrotic tumour volume as computed by (*8*), tumour volume as computed by (*8*), vital tumour volume as computed by (*8*), maximal radius used in the radial filtration, number of vessel loops per vessel segment, median persistence of bars in dimension 2 barcodes (voids), number of short bars per vessel segment in the dimension 0 barcodes. We highlight the topological measures in orange including both the number of loops and number of loops per vessel segment to highlight the effect of the normalisation.

REFERENCES AND NOTES

1. R. Bates, L. Risser, B. Irving, B. W. Papież, P. Kannan, V. Kersemans, J. A. Schnabel, Filling large discontinuities in 3D vascular networks using skeleton- and intensity-based information, in *Medical Image Computing and Computer-Assisted Intervention. MICCAI 2015*, N. Navab, J. Hornegger, W. M. Wells, A. F. Frangi, Eds. (Springer, 2015), vol. 9351 of *Lecture Notes in Computer Science*, pp. 157–164.

2. R. Bates, B. Irving, B. Markelc, J. Kaeppler, G. Brown, R. J. Muschel, M. Brady, V. Grau, J. A. Schnabel, Segmentation of vasculature from fluorescently labeled endothelial cells in multi-photon microscopy images. *IEEE Trans. Med. Imaging* **38**, 1–10 (2019).

3. S. Goel, D. G. Duda, L. Xu, L. L. Munn, Y. Boucher, D. Fukumura, R. K. Jain, Normalization of the vasculature for treatment of cancer and other diseases. *Physiol. Rev.* **91**, 1071–1121 (2011).

4. J. A. Nagy, S. -H. Chang, A. M. Dvorak, H. F. Dvorak, Why are tumour blood vessels abnormal and why is it important to know? *Br. J. Cancer* **100**, 865–869 (2009).

5. M. J. Pittet, R. Weissleder, Intravital imaging. *Cell* **147**, 983–991 (2011).

6. L. Ritsma, E. J. Steller, S. I. Ellenbroek, O. Kranenburg, I. H. M. B. Rinkes, J. Van Rheenen, Surgical implantation of an abdominal imaging window for intravital microscopy. *Nat. Protoc.* **8**, 583–594 (2013).

7. H.-U. Dodt, U. Leischner, A. Schierloh, N. Jährling, C. P. Mauch, K. Deininger, J. M. Deussing, M. Eder, W. Zieglgänsberger, K. Becker, Ultramicroscopy: Three-dimensional visualization of neuronal networks in the whole mouse brain. *Nat. Methods* **4**, 331–336 (2007).

8. M. Dobosz, V. Ntziachristos, W. Scheuer, S. Strobel, Multispectral fluorescence ultramicroscopy: Three-dimensional visualization and automatic quantification of tumor morphology, drug penetration, and antiangiogenic treatment response. *Neoplasia* **16**, 1–13 (2014).

9. G. Vilanova, I. Colominas, H. Gomez, Computational modeling of tumor-induced angiogenesis. *Arch. Comput. Methods Eng.* **24**, 1071–1102 (2017).

10. Y. Gazit, J. W. Baish, N. Safabakhsh, M. Leunig, L. T. Baxter, R. K. Jain, Fractal characteristics of tumor vascular architecture during tumor growth and regression. *Microcirculation* **4**, 395–402 (1997).

11. D. J. Gould, T. J. Vadakkan, R. A. Poché, M. E. Dickinson, Multifractal and lacunarity analysis of microvascular morphology and remodeling. *Microcirculation* **18**, 136–151 (2011).

12. J. Ehling, B. Theek, F. Gremse, S. Baetke, D. Möckel, J. Maynard, S.-A. Ricketts, H. Grüll, M. Neeman, R. Knuechel, W. Lederle, F. Kiessling, T. Lammers, Micro-CT imaging of tumor angiogenesis: Quantitative measures describing micromorphology and vascularization. *Am. J. Clin. Pathol.* **184**, 431–441 (2014).

13. E. Bullitt, D. Zeng, G. Gerig, S. Aylward, S. Joshi, J. K. Smith, W. Lin, M. G. Ewend, Vessel tortuosity and brain tumor malignancy: A blinded study. *Acad. Radiol.* **12**, 1232–1240 (2005).

14. J. Hoffmann, Y. Bar-Sinai, L. M. Lee, J. Andrejevic, S. Mishra, S. M. Rubinstein, C. H. Rycroft, Machine learning in a data-limited regime: Augmenting experiments with synthetic data uncovers order in crumpled sheets. *Sci. Adv.* **5**, eaau6792 (2019).

15. J. Geng, X. Zhang, S. Prabhu, S. H. Shahoei, E. R. Nelson, K. S. Swanson, M. A. Anastasio, A. M. Smith, 3D microscopy and deep learning reveal the heterogeneity of crown-like structure microenvironments in intact adipose tissue. *Sci. Adv.* **7**, eabe2480 (2021).

16. H. Edelsbrunner, D. Letscher, A. Zomorodian, Topological persistence and simplification. *Discrete Comput. Geom.* **28**, 511–533 (2002).

17. G. Carlsson, Topology and data. *Bull. New Ser. Am. Math. Soc.* **46**, 255–308 (2009).

18. H. Edelsbrunner, J. L. Harer, Persistent homology—A survey. *Contemp. Math.* **453**, 257–282 (2008).

19. H. Edelsbrunner, J. L. Harer, *Computational Topology* (American Mathematical Society, 2010).

20. N. Otter, M. A. Porter, U. Tillmann, P. Grindrod, H. A. Harrington, A roadmap for the computation of persistent homology. *EPJ Data Sci.* **6**, 17 (2017).

21. B. J. Stolz, H. A. Harrington, M. A. Porter, Persistent homology of time-dependent functional networks constructed from coupled time series. *Chaos* **27**, 047410 (2017).

22. B. J. Stolz, T. Emerson, S. Nahkuri, M. A. Porter, H. A. Harrington, Topological data analysis of task-based fMRI data from experiments on schizophrenia. *J. Phys. Complex.* **2**, 035006 (2021).

23. A. E. Sizemore, J. E. Phillips-Cremins, R. Ghrist, D. S. Bassett, The importance of the whole: Topological data analysis for the network neuroscientist. *Netw. Neurosci.* **3**, 656–673 (2018).

24. C. Giusti, E. Pastalkova, C. Curto, V. Itskov, Clique topology reveals intrinsic geometric structure in neural correlations. *Proc. Natl. Acad. Sci. U.S.A.* **112**, 13455–13460 (2015).

25. M. W. Reimann, M. Nolte, M. Scolamiero, K. Turner, R. Perin, G. Chindemi, P. Dłotko, R. Levi, K. Hess, H. Markram, Cliques of neurons bound into cavities provide a missing link between structure and function. *Front. Comput. Neurosci.* **11**, 48 (2017).

26. P. Bendich, J. S. Marron, E. Miller, A. Pieloch, S. Skwerer, Persistent homology analysis of brain artery trees. *Ann. Appl. Stat.* **10**, 198–218 (2016).

27. L. Kanari, P. Dłotko, M. Scolamiero, R. Levi, J. Shillcock, K. Hess, H. Markram, A topological representation of branching neuronal morphologies. *Neuroinformatics* **16**, 3–13 (2018).

28. F. Belchi, M. Pirashvili, J. Conway, M. Bennett, R. Djukanovic, J. Brodzki, Lung topology characteristics in patients with chronic obstructive pulmonary disease. *Sci. Rep.* **8**, 5341 (2018).

29. J. Nicponski, J.-H. Jung, Topological data analysis of vascular disease: A theoretical framework. *Front. Appl. Math. Stat.* **6**, 34 (2020).

30. M. R. McGuirl, A. Volkening, B. Sandstede, Topological data analysis of zebrafish patterns. *Proc. Natl. Acad. Sci. U.S.A.* **117**, 5113–5124 (2020).

31. D. Taylor, F. Klimm, H. A. Harrington, M. Kramár, K. Mishchaikow, M. A. Porter, P. J. Mucha, Topological data analysis of contagion maps for examining spreading processes on networks. *Nat. Commun.* **6**, 7723 (2015).

32. M. Feng, M. A. Porter, Spatial applications of topological data analysis: Cities, snowflakes, random structures, and spiders spinning under the influence. *Phys. Rev. Res.* **2**, 033426 (2020).

33. B. J. Stolz-Pretzer, "Global and local persistent homology for the shape and classification of biological data," thesis, University of Oxford (2019).

34. J. L. Nielson, J. Paquette, A. W. Liu, C. F. Guandique, C. A. Tovar, T. Inoue, K.-A. Irvine, J. C. Gensel, J. Kloke, T. C. Petrossian, P. Y. Lum, G. E. Carlsson, G. T. Manley, W. Young, M. S. Beattie, J. C. Bresnahan, A. R. Ferguson, Topological data analysis for discovery in preclinical spinal cord injury and traumatic brain injury. *Nat. Commun.* **6**, 8581 (2015).

35. A. Bukkuri, N. Andor, I. K. Darcy, Applications of topological data analysis in oncology. *Front. Artif. Intell.* **4**, 659037 (2021).

36. J. T. Nardini, B. J. Stolz, K. B. Flores, H. A. Harrington, H. M. Byrne, Topological data analysis distinguishes parameter regimes in the Anderson-Chaplain model of angiogenesis. *PLoS Comput. Biol.* **17**, e1009094 (2021).

37. P. Kannan, W. W. Kretzschmar, H. Winter, D. Warren, R. Bates, P. D. Allen, N. Syed, B. Irving, B. W. Papiez, J. Kaeppler, B. Markelc, P. Kinchesh, S. Gilchrist, S. Smart, J. A. Schnabel, T. Maughan, A. L. Harris, R. J. Muschel, M. Partridge, R. A. Sharma, V. Kersemans, Functional parameters derived from magnetic resonance imaging reflect vascular morphology in preclinical tumors and in human liver metastases. *Clin. Cancer Res.* **24**, 4694–4704 (2018).

38. R. K. Jain, Normalizing tumor microenvironment to treat cancer: Bench to bedside to biomarkers. *J. Clin. Oncol.* **31**, 2205–2218 (2013).

39. S. K. Liu, S. A. Bham, E. Fokas, J. Beech, J. Im, S. Cho, A. L. Harris, R. J. Muschel, Delta-like ligand 4–Notch blockade and tumor radiation response. *J. Natl. Cancer Inst.* **103**, 1778–1798 (2011).

40. J. Folkman, Tumor angiogenesis: Therapeutic implications. *N. Engl. J. Med.* **285**, 1182–1186 (1971).

41. D. Hanahan, R. A. Weinberg, Hallmarks of cancer: The next generation. *Cell* **144**, 646–674 (2011).

42. H. J. Park, R. J. Griffin, S. Hui, S. H. Levitt, C. W. Song, Radiation-induced vascular damage in tumors: Implications of vascular damage in ablative hypofractionated radiotherapy (SBRT and SRS). *Radiat. Res.* **177**, 311–327 (2012).

43. N. Ferrara, K. J. Hillan, H.-P. Gerber, W. Novotny, Discovery and development of bevacizumab, an anti-VEGF antibody for treating cancer. *Nat. Rev. Drug Discov.* **3**, 391–400 (2004).

44. R. Bates, Russ-learn: Set of tools for application and training of deep learning methods for image segmentation and vessel analysis; https://ibme-gitcvs.eng.ox.ac.uk/RussellB/unet-test [accessed 2018].

45. R. Bates, "Learning to extract tumour vasculature: Techniques in machine learning for medical image analysis," thesis, University of Oxford (2017).

46. N. Weidner, Intratumor microvessel density as a prognostic factor in cancer. *Am. J. Pathol.* **147**, 9–19 (1995).

47. H. Edelsbrunner, The union of balls and its dual shape, in *Proceedings of the Ninth Annual Symposium on Computational Geometry* (ACM, 1993), pp. 218–231.

48. B. Delaunay, Sur la sphère vide. A la mémoire de Georges Voronoï. *Bulletin de l'Académie des Sciences de l'URSS. Classe des sciences mathématiques et naturelles*, 793–800 (1934).

49. R. Ghrist, Barcodes: The persistent topology of data. *Bull. New Ser. Am. Math. Soc.* **45**, 61–75 (2008).

50. A. J. Kavran, A. Clauset, Denoising large-scale biological data using network filters. *BMC Bioinformatics* **22**, 157 (2021).

51. D. Cohen-Steiner, H. Edelsbrunner, J. Harer, Stability of persistence diagrams. *Discrete Comput. Geom.* **37**, 103–120 (2007).

52. R. Shayan, T. Karnezis, E. Tsantikos, S. P. Williams, A. S. Runting, M. W. Ashton, M. G. Achen, M. L. Hibbs, S. A. Stacker, A system for quantifying the patterning of the lymphatic vasculature. *Growth Factors* **25**, 417–425 (2007).

53. M. A. Konerding, W. Malkusch, B. Klapthor, C. van Ackern, E. Fait, S. A. Hill, C. Parkins, D. J. Chaplin, M. Presta, J. Denekamp, Evidence for characteristic vascular patterns in solid tumours: Quantitative studies using corrosion casts. *Br. J. Cancer* **80**, 724–732 (1999).

54. P. Breiding, S. Kališnik, B. Sturmfels, M. Weinstein, Learning algebraic varieties from samples. *Rev. Mat. Complut* **31**, 545–593 (2018).

55. L. Crawford, A. Monod, A. X. Chen, S. Mukherjee, R. Rabadán, Predicting clinical outcomes in glioblastoma: An application of topological and functional data analysis. *J. Am. Stat. Assoc.* **115**, 1139–1150 (2020).

56. E. Brown, J. Brunker, S. E. Bohndiek, Photoacoustic imaging as a tool to probe the tumour microenvironment. *Dis. Model. Mech.* **12**, dmm039636 (2019).

57. L. Lin, P. Hu, J. Shi, C. M. Appleton, K. Maslov, L. Li, R. Zhang, L. V. Wang, Single-breath-hold photoacoustic computed tomography of the breast. *Nat. Commun.* **9**, 2352 (2018).

58. H. Adams, T. Emerson, M. Kirby, R. Neville, C. Peterson, P. Shipman, S. Chepushtanova, E. Hanson, F. Motta, L. Ziegelmeier, Persistence images: A stable vector representation of persistent homology. *J. Mach. Learn. Res.* **18**, 1–35 (2017).

59. P. Bubenik, Statistical topological data analysis using persistence landscapes. *J. Mach. Learn. Res.* **16**, 77–102 (2015).

60. P. Bubenik, P. Dłotko, A persistence landscapes toolbox for topological statistics. *J. Symb. Comput.* **78**, 91–114 (2017).

61. Y. Wang, H. Ombao, M. K. Chung, Topological seizure origin detection in electroencephalographic signals, in *IEEE 12th International Symposium on Biomedical Imaging (ISBI)* (2015), pp. 351–354.

62. V. Kovacev-Nikolic, P. Bubenik, D. Nikolić, G. Heo, Using persistent homology and dynamical distances to analyze protein binding. *Stat. Appl. Genet. Mol. Biol.* **15**, 19–38 (2016).

63. A. Garg, D. Lu, K. Popuri, M. F. Beg, Brain geometry persistent homology marker for parkinson's disease, in *IEEE 14th International Symposium on Biomedical Imaging* (ISBI, 2017), pp. 525–528.

64. O. Vipond, J. A. Bull, P. S. Macklin, U. Tillmann, C. W. Pugh, H. M. Byrne, H. A. Harrington, Multiparameter persistent homology landscapes identify immune cell spatial patterns in tumors. *Proc. Natl. Acad. Sci. U.S.A.* **118**, e2102166118 (2021).

65. R. Penta, D. Ambrosi, The role of the microvascular tortuosity in tumor transport phenomena. *J. Theor. Biol.* **364**, 80–97 (2015).

66. M. Hill, J. Thompson, A. Kavanagh, I. Tullis, R. Newman, J. Prentice, J. Beech, S. Gilchrist, S. Smart, E. Fokas, B. Vojnovic, The development of technology for effective respiratory-gated irradiation using an image-guided small animal irradiator. *Radiat. Res.* **188**, 247–263 (2017).

67. C. A. Hudis, Trastuzumab—Mechanism of action and use in clinical practice. *N. Engl. J. Med.* **357**, 39–51 (2007).

68. W. Rasband, ImageJ. Image processing and analysis in JAVA: https://imagej.nih.gov/ij/download.html [accessed 2019].

69. Sainsbury Wellcome Centre, MATLAB toolbox for analysis of output from the software aMAP (optimized automated mouse atlas propagation) [accessed 2019]. Toolbox available at www.gatsby.ucl.ac.uk/-test/matlabTools.zip. See https://github.com/SainsburyWellcomeCentre/aMAP/wiki for description.

70. J. R. Munkres, *Topology* (Pearson Prentice Hall, 2000).

71. C. M. Topaz, Self-help homology tutorial for the simple(x)-minded (2015); https://drive.google.com/file/d/0B3Www1z6Tm8xV3ozTmN5RE94bDg/view, [accessed 8 August 2019].

72. C. Kosniowski, *A First Course in Algebraic Topology* (Cambridge Univ. Press, 1980).

73. A. Hatcher, *Algebraic topology* (Cambridge Univ. Press, 2001).

74. J. R. Munkres, *Elements of Algebraic Topology* (The Benjamin/Cummings Publishing Company Inc., 1984).

75. V. Rouvreau, Alpha complex. *GUDHI User and Reference Manual* (GUDHI Editorial Board, ed. 3.1.1, 2020).

76. RStudio Team, *RStudio: Integrated Development Environment for R* (RStudio Inc., 2016).

77. A. Tausz, M. Vejdemo-Johansson, H. Adams, JavaPlex: A research software package for persistent (co)homology. Mathematical Software. ICMS 2014, H. Hong, C. Yap, Eds. (Springer, 2014), vol. 8592 of Lecture Notes in Computer Science, pp. 129–136: http://appliedtopology.github.io/javaplex/ [accessed 2017].

78. C. Maria, J.-D. Boissonnat, M. Glisse, M. Yvinec, International congress on mathematical software (Springer, 2014), pp. 167–174: https://gudhi.inria.fr [accessed 2020].

79. G. Carlsson, A. Zomorodian, A. Collins, L. J. Guibas, Persistence barcodes for shapes. *Int. J. Shape Model.* **11**, 149–187 (2005).

80. A. Zomorodian, G. Carlsson, Computing persistent homology. *Discrete Comput. Geom.* **33**, 249–274 (2005).
